# qtlXplorer: an online systems genetics browser in the *Eucalyptus* Genome Integrative Explorer (EucGenIE)

**DOI:** 10.1186/s12859-021-04514-9

**Published:** 2021-12-15

**Authors:** Nanette Christie, Chanaka Mannapperuma, Raphael Ployet, Karen van der Merwe, Niklas Mähler, Nicolas Delhomme, Sanushka Naidoo, Eshchar Mizrachi, Nathaniel R. Street, Alexander A. Myburg

**Affiliations:** 1 Department of Biochemistry, Genetics and Microbiology, Forestry and Agricultural Biotechnology Institute (FABI), University of Pretoria, Private bag X20, Pretoria, 0028 South Africa; 2 Umeå Plant Science Centre, Department of Plant Physiology, Umeå University, 907 81 Umeå, Sweden; 3 Umeå Plant Science Centre, Department of Forest Genetics and Plant Physiology, Swedish University of Agricultural Sciences, 901 83 Umeå, Sweden

**Keywords:** qtlXplorer, *Eucalyptus*, EucGenIE, Systems genetics, eQTL, Co-expression, ‘Omics integration, Online resource, Database, Genome browser

## Abstract

**Background:**

Affordable high-throughput DNA and RNA sequencing technologies are allowing genomic analysis of plant and animal populations and as a result empowering new systems genetics approaches to study complex traits. The availability of intuitive tools to browse and analyze the resulting large-scale genetic and genomic datasets remain a significant challenge. Furthermore, these integrative genomics approaches require innovative methods to dissect the flow and interconnectedness of biological information underlying complex trait variation. The Plant Genome Integrative Explorer (PlantGenIE.org) is a multi-species database and domain that houses online tools for model and woody plant species including *Eucalyptus*. Since the *Eucalyptus* Genome Integrative Explorer (EucGenIE) is integrated within PlantGenIE, it shares genome and expression analysis tools previously implemented within the various subdomains (ConGenIE, PopGenIE and AtGenIE). Despite the success in setting up integrative genomics databases, online tools for systems genetics modelling and high-resolution dissection of complex trait variation in plant populations have been lacking.

**Results:**

We have developed qtlXplorer (https://eucgenie.org/QTLXplorer) for visualizing and exploring systems genetics data from genome-wide association studies including quantitative trait loci (QTLs) and expression-based QTL (eQTL) associations. This module allows users to, for example, find co-located QTLs and eQTLs using an interactive version of Circos, or explore underlying genes using JBrowse. It provides users with a means to build systems genetics models and generate hypotheses from large-scale population genomics data. We also substantially upgraded the EucGenIE resource and show how it enables users to combine genomics and systems genetics approaches to discover candidate genes involved in biotic stress responses and wood formation by focusing on two multigene families, laccases and peroxidases.

**Conclusions:**

qtlXplorer adds a new dimension, population genomics, to the EucGenIE and PlantGenIE environment. The resource will be of interest to researchers and molecular breeders working in *Eucalyptus* and other woody plant species. It provides an example of how systems genetics data can be integrated with functional genetics data to provide biological insight and formulate hypotheses. Importantly, integration within PlantGenIE enables novel comparative genomics analyses to be performed from population-scale data.

**Supplementary Information:**

The online version contains supplementary material available at 10.1186/s12859-021-04514-9.

## Background

Systems genetics approaches aim to understand how molecular components interact to determine the complex relationships between gene networks and traits [1]. In contrast to systems biology, which typically investigates biological systems in a common genetic background, the focus shifts to studying populations of genetically diverse individuals [2]. One of the challenges to exploring large systems genetics data sets, *e.g.* in genome-wide association analyses such as expression quantitative trait locus (eQTL) mapping, is the integration and visualization of different data layers [3]. Compared to systems biology resources, there is a lack of databases with an integrated suite of tools for plant systems genetics. For systems genetics studies, new database structures, queries and visualization tools are required to effectively interrogate the data with a focus on genetic variation linked to molecular traits such as transcript and metabolite variation.

As in other biological fields, the state-of-the-art in plant genomics research is shifting towards integrative, systems-level analyses of biological processes. Online genomics resources have been developed to cope with the large amounts of ‘omics data now available for many plant species and to provide researchers access to web-based tools for online analysis and visualization of increasingly complex ‘omics datasets [4]. Online platforms such as Phytozome [5], Ensembl Plants [6], Gramene [7] and PLAZA [8] provide broad taxonomic coverage, while others such as The *Arabidopsis* Information Resource (TAIR) [9] and the Maize Genetics and Genomics Database (MaizeGDB) [10] provide access to deep genomics and genetics information for individual model species. Some online resources such as the Bio-Analytic Resource (BAR) [11] and the Plant Genome Integrative Explorer (PlantGenIE) [12] have specialized in online browsing and visualization of plant transcriptome data, using tools such as electronic Fluorescent Pictograph (eFP) [13] and Expression Angler [14]. Another example, Genevestigator [15], is an online transcriptome analysis resource that is highly curated and commercialized, while PlaNet [16] was designed to transfer knowledge across species via conserved co-expression networks and sequence relationships.

Wood from plantation forestry is a renewable feedstock for bio-based materials (timber, pulp and paper) and energy and thus will increasingly contribute to the transition from a fossil carbon economy to a more sustainable bio-based economy [17]. Furthermore, woody plants have a crucial role in global carbon sequestration. A recent study in climate change ecology has suggested that a global effort to plant one trillion trees would sequester sufficient carbon to remove two thirds of anthropogenic CO_2_ (~205 Gt of ~300 Gt) from the atmosphere [18]. However, over 200 million ha, mostly in the tropics, are under threat and may disappear by 2050. A challenge in woody plant biology research is therefore to understand climate adaptation in forest trees and, in the case of plantation forestry, to produce more wood with improved properties from a shrinking land base. Even though forest tree genomes generally are highly diverse and outbred and, in the case of conifers, highly complex and large (19-22 Gbp) [19], excellent resources have been developed to meet the needs of the forest tree research community, supporting data from various ‘omic layers, as well as analysis and cross-site queries in a web-based environment [20]. These resources are becoming part of integrated content management systems (CMS) that support specific, shareable modules for query and analysis. For example, TreeGenes [21], Hardwood Genomics Project [22] and Genome Database for *Rosaceae* [23] recently migrated to Tripal [24], and PlantGenIE [12] has been updated to Genome Integrative Explorer System (GenIE-Sys) [25].

The Plant Genome Integrative Explorer (PlantGenIE) web resource [12] has a primary focus on tools for exploring forest tree genomics and transcriptomics data from model woody angiosperm (*Populus*, PopGenIE) and conifer (*Picea*, ConGenIE) systems. The PlantGenIE.org umbrella platform consists of a common set of tools that are available across species and the architecture facilitates the addition of new analysis and browsing tools for visualizing gene expression profiles from RNA-sequencing (RNA-seq) datasets (exImage, exPlot and exHeatmap), exploring co-expression networks (exNet), comparative co-expression network conservation (ComPlEx) [26] and testing for functional category enrichment (Enrichment). Additional gene expression resources for aspen (AspWood) [27] and Norway spruce (NorWood) [28] comprising high-spatial-resolution transcriptome profiles during wood formation are being integrated within the PopGenIE and ConGenIE sites, respectively.

*Eucalyptus* species and hybrids comprise the most widely cultivated hardwood fibre crop (over 20 million ha of plantations globally), which has become an important renewable feedstock for lignocellulosic products and fiber for pulp and paper production. The completion of a reference genome [29], subsequent transcriptomics studies [30] and development of genome-wide genotyping resources [31] has produced a rapidly growing base of genomics and genetic data. The first version of EucGenIE [32] was developed to host an early transcriptome assembly and RNA-seq datasets from a range of developing tissues [33] and, after completion of the reference genome, many other transcriptomes from xylogenic [34], photosynthetic and reproductive tissues [35], as well as pest and disease responses [36–38] were added.

Here we describe qtlXplorer, a new tool for browsing systems genetics data, as part of a remodelled version of the EucGenIE resource. EucGenIE was recently implemented within PlantGenIE (via the GenIE-Sys) and is a dedicated platform for the exploration of *Eucalyptus* genomics data. Together with the existing PlantGenIE tools, qtlXplorer provides a means to generate hypotheses from evidence of co-expression and co-regulation in large-scale population genomics data. To illustrate the features and capability of qtlXplorer within EucGenIE, we present a case study investigating the roles of laccases and peroxidases in biotic stress response and wood formation in *Eucalyptus*. We combined systems biology and systems genetics analyses to prioritize the best candidate laccase and peroxidase genes involved in lignin polymerization and highlight the genetic architecture of their regulation in the form of a systems genetics model.

## Construction and content

**Fig. 1:**
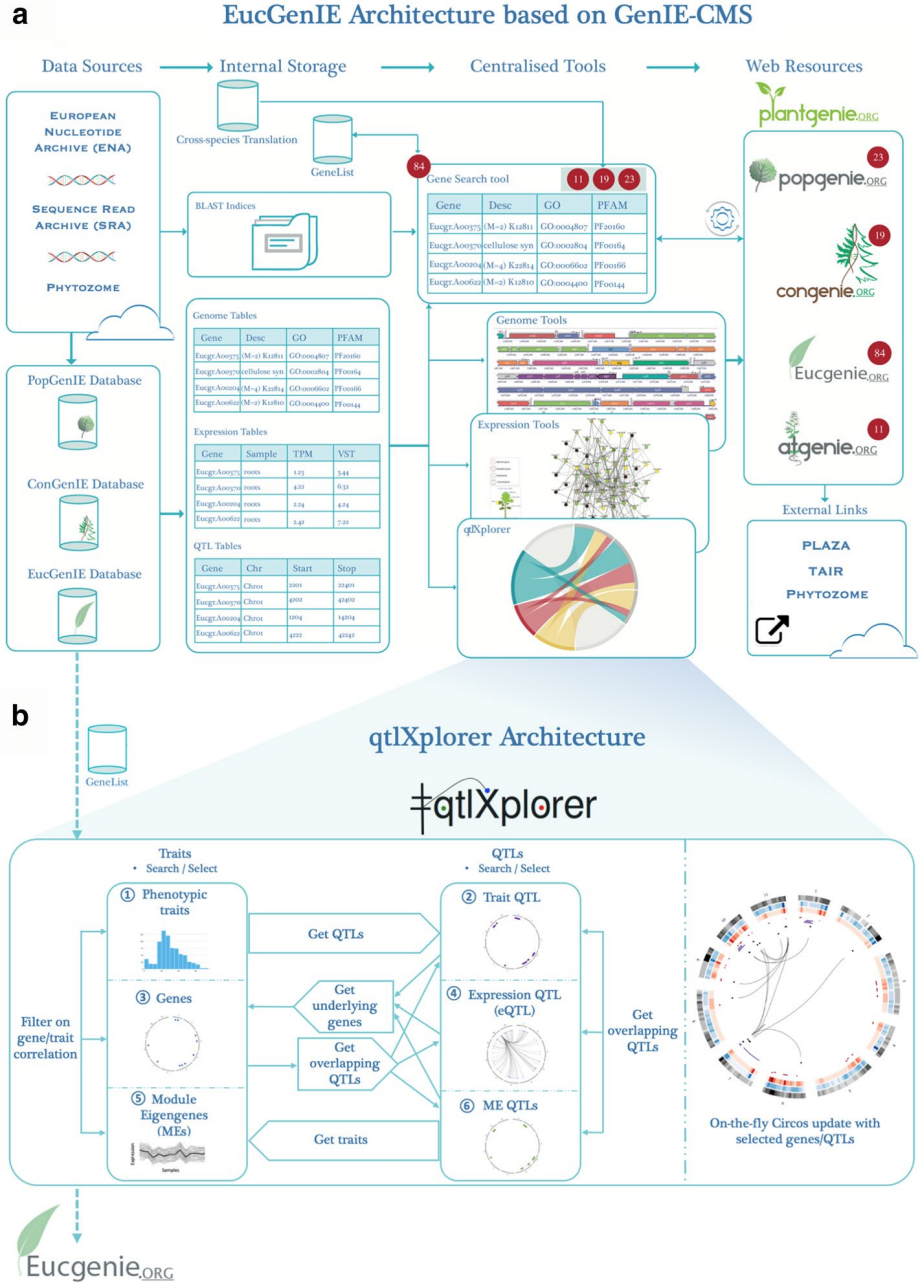
Overview of the *Eucalyptus* Genome Integrative Explorer (EucGenIE) and qtlXplorer architecture. **a** EucGenIE integrates data from various sources and makes it available for query and exploration via a set of centralized plugins, e.g. interactive visualisation tools within the Genome Integrative Explorer System (GenIE-Sys). The Plant Genome Integrative Explorer (http://PlantGenIE.org) domain serves as an umbrella site linking four species-specific subdomains including EucGenIE.org. The databases contain tables for genomic, expression and QTL data from various sources. The Gene Search tool queries data from the genome table, enabling the user to search the database using gene identifiers or free-text search terms to create gene lists. A user can subsequently select tools to explore expression and annotation data linked to the genes in the active gene list, or create cross-species gene lists using orthology inference methods to move between different PlantGenIE domains. In the example presented, 84 laccase-related *Eucalyptus* genes were identified using the free text search term ‘laccase’. This corresponds to 23 poplar, 19 spruce and 11 *Arabidopsis* orthologs. PlantGenIE has external links to different web resources such as PLAZA, TAIR and Phytozome, to enable further downstream, in-depth exploration of biological data. **b** qtlXplorer is the first population genomics tool for the exploration of systems genetics data, currently only implemented in EucGenIE.org. There are six different starting points (1–6) for querying and browsing the data on an interactive Circos plot updating in real-time. Starting points 3 and 4 can also include other component traits (e.g. metabolites and metabolite QTLs) and starting points 5 and 6 can also include eigentraits or eigenmetabolites with the corresponding eigenQTLs. Each starting point consists of a searchable and sortable table from where a user can select instances. Input to qtlXplorer can be new or existing gene lists from the Gene Search tool in EucGenIE. Gene lists can be exported for further exploration in EucGenIE

We developed qtlXplorer as the first population genomics tool in the PlantGenIE.org environment. qtlXplorer is available as a tool to browse *Eucalyptus* systems genetic data in EucGenIE, which is one of the four species-specific subdomains of the online PlantGenIE.org umbrella platform (Fig. 1a). In contrast to other EucGenIE tools that focus on analysis and visualization of gene expression data across tissues and environmental perturbations, qtlXplorer enables mining profiles of quantitative traits such as wood growth, wood composition, and gene transcript levels, across individuals from a population; and exploring regions in the genome that affect variation in these traits and genes (trait QTLs and eQTLs).

Currently qtlXplorer allows exploration of transcriptomic data for 283 samples from two *E. grandis* x *E. urophylla* backcross populations (sample collection 1 in Additional file 1: Table S1). The population-wide transcriptome data was used to map eQTLs, compute gene pairwise correlations and identify co-expression modules. For each co-expression module, a module eigengene (ME; weighted summary profile representing that module) was calculated and ME QTLs were mapped. In addition, population-wide phenotype data (wood property and growth traits) was used to map trait QTLs. Additional file 2: Method S1 provides detail on the expression profiling of the population transcriptome datasets and Additional file 2: Method S2 on the trait QTL and eQTL mapping and analysis steps in the data processing pipeline [39]. As a result, trait QTL and eQTL data are available for four population-map combinations: (1)* E. urophylla* backcross—F1 Hybrid map, (2) *E. urophylla* backcross—backcross parent map, (3) *E. grandis* backcross—F1 Hybrid map and (4) *E. grandis* backcross—backcross parent map.

qtlXplorer provides the user with six different panels (starting points) for browsing and querying the available systems genetics data. These include phenotypic traits, trait QTLs, genes, eQTLs, MEs and ME QTLs (Fig. 1b). Gene and QTL annotations are provided, including gene co-expression module membership, *trans*-eQTL hotspot membership and eQTL classification as *cis* or *trans* (Additional file 2: Method S2). Each panel consists of a searchable and sortable table from which a user can select various items to use as a basis for QTL angling. For example, after importing a gene list, a user can (i) get the eQTLs mapped for the query genes, (ii) get QTLs that overlap (co-localize) with the genomic positions of these genes or (iii) filter to keep only genes with a high, user-provided cut-off, gene–gene correlation (co-expression). In addition, after selecting a set of eQTLs from the eQTL panel, a user can (iv) save the target genes to a gene list (for further exploration within EucGenIE), (v) get genes that underlie the query eQTLs or (vi) get eQTLs or trait QTLs that overlap (co-localize) with the genomic position of the query eQTLs (Fig. 1b). Each query will redirect the user to the relevant panel where the results can be exported as a tabulated file, and from where a follow-up query will be possible.

In a parallel view, the genomic positions of genes and QTLs (eQTLs, trait QTLs and ME QTLs) are displayed on-the-fly, on a Circos plot [40] (Fig. 1b). A user can switch between instances of QTLs by changing the selection in the table. qtlXplorer was developed in Java, using Google Web Toolkit (GWT) [41]. The web-based front-end communicates with the database via PHP and data is sent in JavaScript Object Notation (JSON) format. The configuration file for the interactive Circos is created based on the selected items and then executed and linked to an image map file (https://accessibility.psu.edu/imagemaps/). This links the areas on the image to the JBrowse instance in EucGenIE. Therefore, selecting a QTL of interest on the interactive Circos plot will open the EucGenIE JBrowse tool—making it possible to explore and browse the underlying genes.

In addition to qtlXplorer, as mentioned above, EucGenIE comprises an array of generic tools provided by the GenIE-Sys that are available in each of the PlantGenIE resources. These tools have been integrated as centralized groups of tools, which include Gene Search tools, Genome tools, Analysis tools, Expression tools and Population Genomics tools (Table 1). Additional file 2: Method S3 gives an overview of the EucGenIE tools and the case study below provides an illustration of how these tools can be used to explore genomic data. Sample collections for expression data currently hosted in EucGenIE include (Additional file 1: Table S1): (1) *E. grandis* x *E. urophylla* population transcriptomes data, (2) the *E. grandis* tissues exAtlas, (3) *the E. grandis* x *E. urophylla* tissues exAtlas and (4) biotic interactions data. Data in the last three sample collections can be explored with the Expression tools (Table 1; Additional file 2: Method S1 gives detail on expression profiling of these RNA-seq datasets) in EucGenIE, while the first sample collection is reserved for systems genetics analyses with qtlXplorer. Annotation, gene expression and QTL data are stored in the GenIE-Sys database, while DNA sequence data, after conversion, is stored as BLAST and JBrowse indices (Fig. 1a). There are two additional databases, one for user-created gene lists and one for translating gene lists across the set of species available in PlantGenIE. The "translation" database contains all the ortholog information of the plant species included in PlantGenIE, to enable users to move between different tools and subdomains (Additional file 2: Method S4).

**Table 1:** Overview of the analysis and visualization tools available in EucGenIE

Category / Tool name	Description	Tool status
*Gene Search tools*		
GeneList	Search and save genes to a gene list	Customized for EucGenIE
Cross-species GeneList	Create a PlantGenIE (cross-species) gene list using ortholog information	New tool
*Genome tools*		
BLAST	Performs sequence homology searches	GenIE-Sys
JBrowse	Browse genomics data on genome browser	GenIE-Sys
ChrDiagram	Plots the location of genes in the active gene list in chromosomes	GenIE-Sys
SeqSearch	Extracts sequence information for genes in the active gene list	Improved (GenIE-Sys)
*Expression tools*		
exNet	Displays co-expression networks based on precalculated transcriptional expression networks	Improved (GenIE-Sys)
exImage	Visualizes single gene expression profiles using an electronic fluorescent pictograph (eFP)	Improved (GenIE-Sys) Customized for EucGenIE
exPlot	Generates line graphs of expression profiles across samples in the selected experiment	GenIE-Sys
exHeatmap	Represents expression profiles for genes in the active gene list using a heatmap representation	Improved (GenIE-Sys)
exMatch	Finds groups of genes with the desired expression profile	New tool
*Analysis tools*		
Venn	Generates an interactive Venn diagram of the gene lists stored in EucGenIE	New tool
Enrichment	Performs enrichment analysis for gene ontology (GO) categories, Kyoto Encyclopedia of Genes and Genomes (KEGG) pathways and Protein family (Pfam) domains within a gene list	Improved (GenIE-Sys)
*Population Genomics tools*		
qtlXplorer	Performs on-the-fly systems genetics data queries using an interactive version of Circos	New tool

## Utility and discussion

### Case study introduction: laccases and peroxidases

Wood secondary cell walls (SCW) constitute a major biomass feedstock for a variety of lignocellulosic products, including a new generation of bio-based chemicals and biomaterials. However, conversion of lignocellulosic biomass to cellulose-based products is severely impaired by lignin, a phenolic polymer responsible for the recalcitrance of SCWs. Despite extensive work on the lignin biosynthetic pathway in biomass crops [42, 43], the genes encoding the specific enzymes required for the last step of lignin biosynthesis (polymerization of monolignols) remain unknown in *Eucalyptus*. Several members of the two multigenic families of apoplastic oxidases, laccases (LAC) and Class III peroxidases (PRX), have been implicated in radical-coupling of monolignols leading to lignin polymerization in different plant species [44–47]. As key actors in reactive oxygen species (ROS) homeostasis, these two protein families have also been associated with biotic stress responses [48, 49], including ROS scavenging, ROS signalling, apoplastic oxidative burst and synthesis of bioactive secondary metabolites [50, 51]. In this study, we demonstrate how the genomic and systems genetics tools available in EucGenIE can be used to prioritize a set of LAC and PRX genes potentially involved in SCW biosynthesis, and also highlight several LAC and PRX genes likely involved in biotic stress responses in *Eucalyptus*.

### Candidate gene discovery by browsing genomics data via interactive visualization tools in EucGenIE

The GeneList tool is the central function that allows searching and filtering for genes based on different types of annotation and subsequently saves custom gene lists. Using the GeneList tool starting with the keyword “laccase”, in combination with external genomic tools (see Additional file 2: Method S5), we identified a set of 44 genes coding for putative LAC genes likely to be functional and targeted to the apoplast, of which 34 were previously annotated in *E. grandis* by a comprehensive study (Additional file 1: Table S2) [52]. Ninety-three Class III PRX coding genes previously annotated in *E. grandis* (http://peroxibase.toulouse.inra.fr/) [53] were also added to the active gene list, creating a gene list of 137 LAC and PRX genes.

**Fig. 2:**
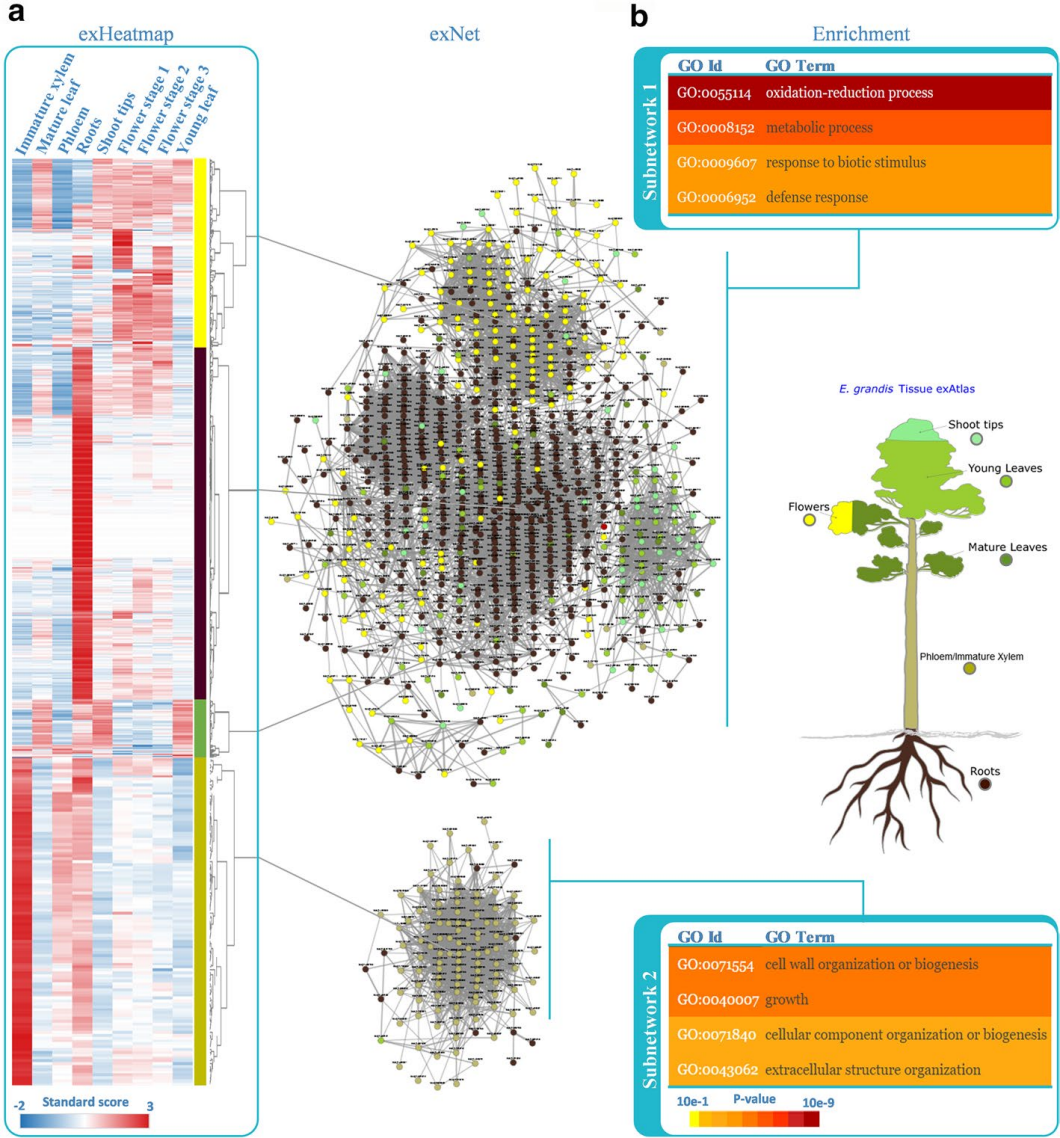
Co-expression analysis of the LAC and PRX genes based on EucGenIE datasets. **a** Visualization of the co-expression network using exNet (default threshold = 5) and expression clusters using exHeatmap, for the initial set of LAC/PRX candidate genes; **b** GO enrichment analysis of the two main subnetworks using Enrichment. The exNet tool allowed the generation of a co-expression network based on 72 transcriptomic datasets across different tissues (exAtlas) and biotic conditions (interaction with *Chrysoporthe austroafricana*, *Phytophthora cinnamomi* and *Leptocybe invasa*). Nodes are coloured according to gene preferential expression across the six tissues in *E. grandis* x *E. urophylla* tissues (exAtlas) and edge thickness is proportional to the absolute correlation value. Heatmaps were created using the exHeatmap tool to visualize standardized values of absolute expression (VST) of genes across the nine tissues in the *E. grandis* tissues in exAtlas. Clustering of genes is based on the Ward method [79] and clusters are related to the main subnetworks. Biological process GO enrichment tests were performed using the Enrichment tool and coloured according to enrichment significance

To associate subsets of the 137 LAC/PRX genes with biological functions, we performed a co-expression analysis using the exNet tool, which allows the visualization of gene profile correlations across the 72 expression datasets available in EucGenIE (sample collections 2–4 in Additional file 1: Table S1; Additional file 3: Figure S1a). exNet also allows expansion of the initial network to identify neighbouring genes directly co-expressed with candidate genes at a certain threshold, and subnetworks (or specific nodes) can then manually be selected to create new gene lists for further analysis (Additional file 3: Figure S1b). The expanded network of the 137 LAC/PRX genes resulted in two main subnetworks (Fig. 2) that were saved as separate gene lists to investigate their expression profile and enrichment for biological functions using exHeatmap and Enrichment tools, respectively. The subnetwork 1 contained 51 LAC/PRX genes showing preferential expression in various tissues (Fig. 2a) including roots (brown), flowers (yellow) and leaves (green), while the second smaller subnetwork (subnetwork 2) contained eight LAC/PRX genes (Additional file 1: Table S3), all preferentially expressed in lignified tissues: phloem/immature xylem (tan) and to a lesser extent roots (Fig. 2a). Subnetwork 1 was significantly enriched for gene ontology (GO) terms related to biotic stress response (defense response, oxidation–reduction and metabolic process; Fig. 2b), while subnetwork 2 showed enrichment for GO terms related to plant development, more specifically cell wall development (cell wall organization or biogenesis, extracellular and cellular organization, and growth; Fig. 2b).

**Fig. 3:**
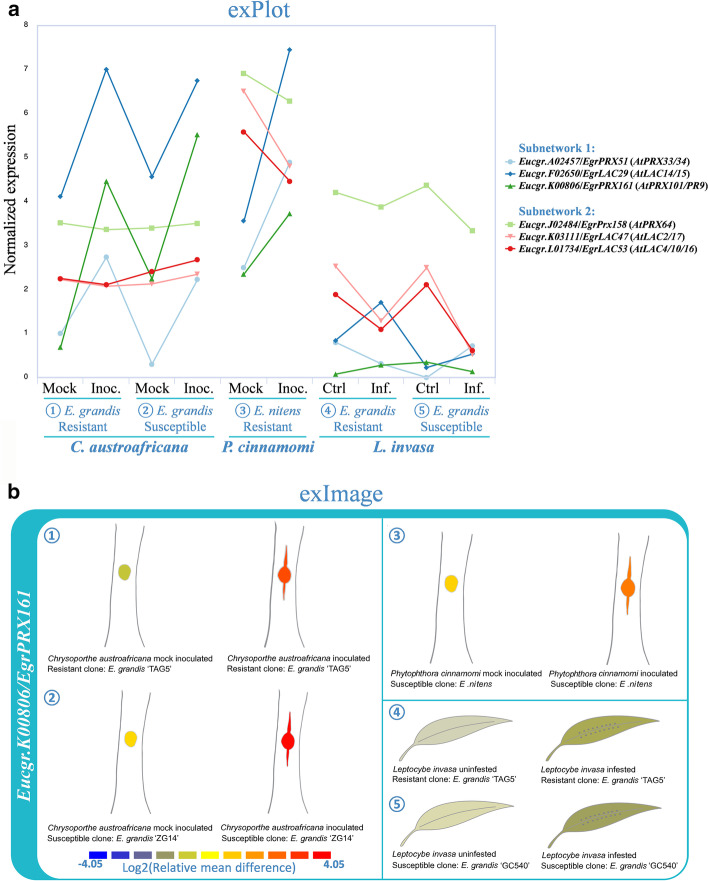
Expression profiles of LAC/PRX genes in response to biotic stress in *Eucalyptus*. **a** Expression profiles of three LAC/PRX genes selected out of 51 genes in subnetwork 1 (significantly enriched for stress-related genes; Fig. 2b) and three of the eight LAC/PRX genes in subnetwork 2 (significantly enriched in SCW-related genes; Fig. 2b) visualized using exPlot; **b** example of exImage output for the gene Eucgr.K00806/EgrPrx161 from subnetwork 1. exPlot and exImage tools allowed the visualization of the normalized expression data (TPM values) and relative expression to the mean (log_2_(fold change)) respectively. Additional file 1: Table S1 provides a summary of the experimental information of the different biotic interaction experiments and relative transcript levels are provided in Additional file 1: Table S3

For hypothesis formulation regarding candidate gene function, more detailed expression profiles across tissues and environmental perturbations can be visualized using exPlot and exImage tools. exPlot displays expression profiles of a compendium of genes as a line chart, while exImage displays eFP browser type plots for individual genes that can be selected interactively. As illustrated for three genes in Fig. 3a (exPlot) and one gene in Fig. 3b (exImage), most of the 51 LAC/PRX genes from the stress-enriched subnetwork 1 were found induced in leaves or stem of *Eucalyptus* seedlings infected by fungal (*C. austroafricana*) or oomycete (*P. cinnamomi*) pathogens, or in response to insect (*L. invasa*) infestation. This included 10 putative orthologs of genes characterized for their function in stress response in various species, as well as 13 genes previously found induced in stress conditions in *Eucalyptus* (Additional file 1: Table S3). In contrast, the majority of the stem preferentially expressed LAC/PRX genes from subnetwork 2 remained unaffected or were repressed in response to biotic stress as illustrated in Fig. 3a.

**Fig. 4:**
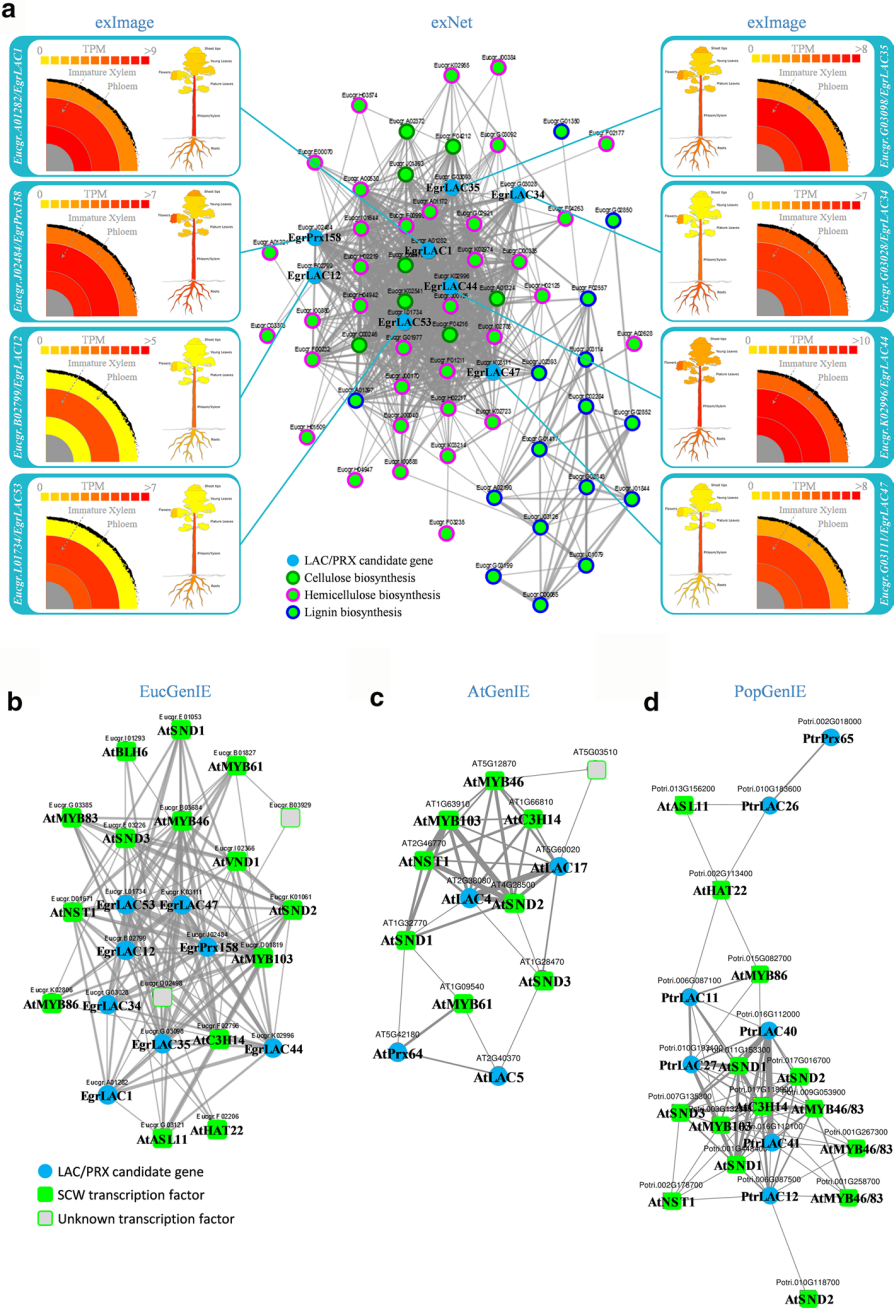
Co-expression of LAC/PRX genes with secondary cell wall (SCW) biosynthesis-related genes. **a** exNet was used to analyse the co-expression of the eight LAC/PRX genes from subnetwork 2 (Fig. 2, Additional file 1: Table S3) specifically with 59 genes involved in the biosynthesis of cellulose, hemicellulose and lignin (Additional file 1: Table S4). exImage allowed the visualization of their detailed absolute expression profiles (TPM) across *Eucalyptus* tissues. **b** Co-expression of the eight candidate genes, with 14 TFs that are putative orthologs of SCW-biosynthesis regulators in *Arabidopsis* and two TFs of unknown function in *Eucalyptus* (Additional file 1: Table S4) (EucGenIE.org); **c** Co-expression of four putative orthologs of the eight *Eucalyptus* LAC/PRX genes (AtLAC4, AtLAC5, AtLAC17 and AtPRX64), with eight TFs involved in the regulation of SCW biosynthesis and one TF of unknown function in *Arabidopsis* (AtGenIE.org); **d** Co-expression of seven putative orthologs of the eight *Eucalyptus* LAC/PRX genes in *Populus*, with 14 TF homologs of *Arabidopsis* SCW regulators (PopGenIE.org). The best diamond BLAST hit option available in the GeneList tool allowed the identification of the putative orthologs of the eight candidate genes in the different GenIE domains (from EucGenIE to AtGenIE and PopGenIE), and exNet was used to generate and visualize the co-expression networks

Furthermore, to test the hypothesis that the eight LAC/PRX genes from subnetwork 2 could be involved in stem development, and investigate a possible role in SCW formation, we again employed exNet and exImage tools. The aims were to i) test the eight LAC/PRX genes’ co-expression specifically with SCW biosynthesis genes previously annotated in *E. grandis* [29, 54] and ii) visualize their detailed expression profile in stem tissues. In the network obtained from exNet, the eight genes were part of a co-expression network with 59 SCW biosynthesis genes, including 35 genes involved in xylan biosynthesis, 16 in lignin biosynthesis and eight in cellulose biosynthesis (Fig. 4a; Additional file 1: Table S4). Within stem tissues (phloem, immature xylem, mature xylem), all eight candidate genes showed preferential expression in immature xylem (Fig. 4a).

This SCW network was expanded and 16 transcription factors (TFs) were identified in the neighbourhood of the eight candidate genes (Fig. 4b, Additional file 1: Table S4). These included known regulators of SCW formation process in *Eucalyptus*, such as *EgMYB2* and its paralog *EgMYB31* (respectively *Eucgr.G03385*, ortholog of *AtMYB83*, and *Eucgr.B03684*, ortholog of *AtMYB46*) [55], as well as *EgMYB137* (*Eucgr.K02806*) [56], and 11 closest orthologs of key regulators of SCW biosynthesis in *Arabidopsis* (AtNST1, AtNST2, AtVND1, AtSND2, AtSND3, AtMYB61, AtMYB103, AtASL11, AtC3H14, AtBLH6, HAT22) [57] (Fig. 4b).

Comparative analysis across species can also be used to refine hypotheses by taking advantage of datasets available in other plant species. From the Cross-species GeneList tool, the best diamond hit option allowed the selection of closest orthologs of the eight LAC/PRX candidates in other plant species and the creation of a PlantGenIE gene list to study their conserved co-expression pattern with SCW TFs in the herbaceous model plant *Arabidopsis* (Fig. 4c) and in another tree species *Populus* (Fig. 4d). Briefly, this approach revealed that the closest orthologs of these *Eucalyptus* genes identified in *Arabidopsis* (*AtLAC4*, *AtLAC17*, *AtLAC5* and *AtPRX64*), all known to be involved in cell wall formation [58–62] are also correlated to SCW regulators (Fig. 4c). Similarly in poplar, four of the putative orthologs of the LAC genes (*PtrLAC26, PtrLAC27*, *PtrLAC40* and *PtrLAC41*) were previously related to cell wall formation [63, 64], including PtrLAC27 functionally associated with lignin biosynthesis [65, 66], and all were directly correlated to the orthologs of regulators of SCW formation (Fig. 4d).

Accordingly, this approach showed that combining the co-expression and expression profile visualization tools to analyse the large amount of transcriptomic data available in EucGenIE (as well as the other domains of PlantGenIE), allowed narrowing down the roles of the initial list of LAC/PRX genes. From the 137 candidate genes, eight are likely dedicated primarily to SCW formation during secondary xylem development in *Eucalyptus*, while the majority of the remaining genes, gathered in subnetwork 1, tend to be associated with stress response in different tissues.

### Systems genetics analysis of candidate genes using qtlXplorer

**Fig. 5:**
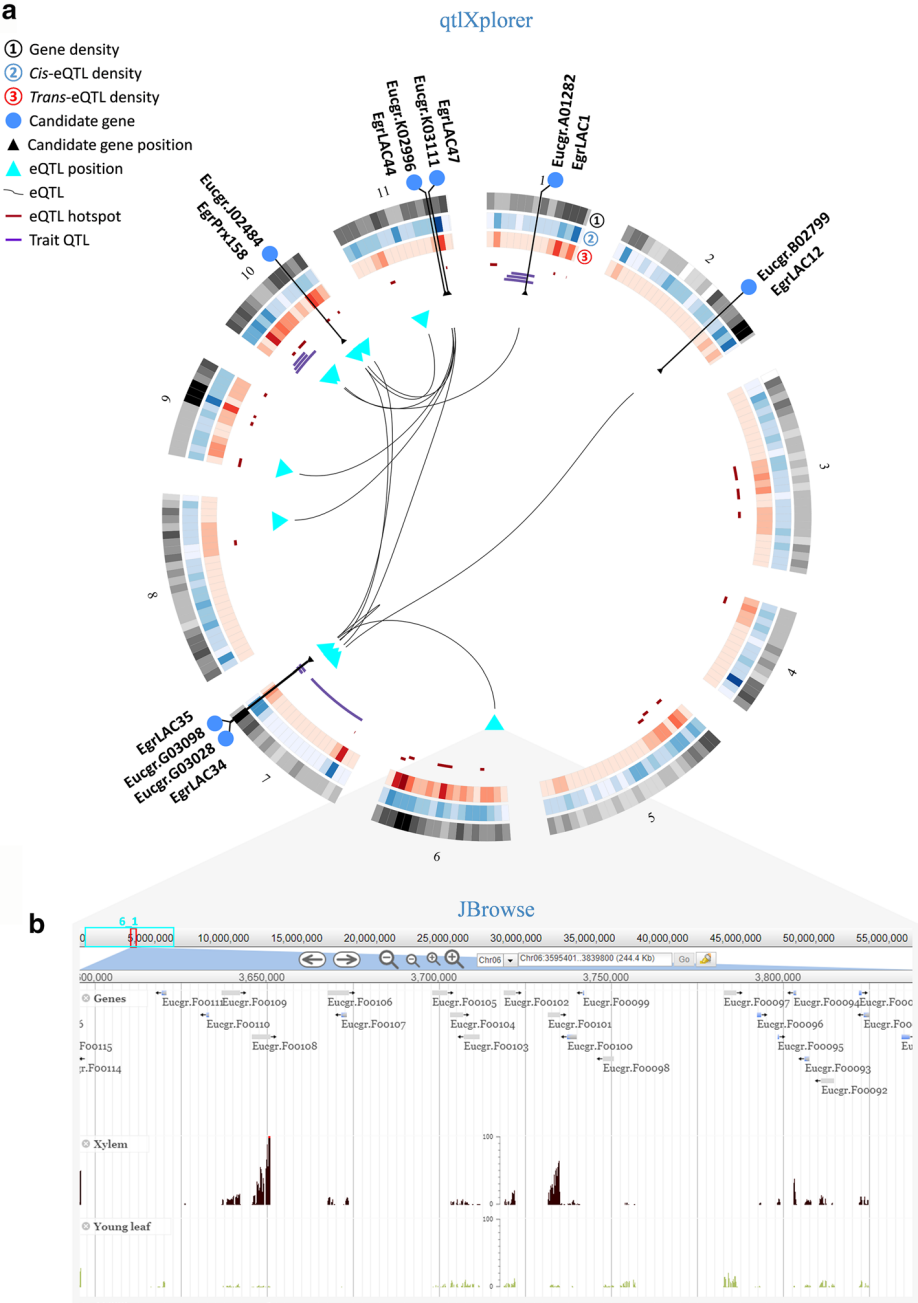
eQTL associations and trait QTL overlap of eight SCW-related LAC/PRX genes visualized using qtlXplorer. **a** Circos plot representation of the genomic positions of seven out of the eight LAC/PRX candidates from subnetwork 2 (Fig. 2, Additional file 1: Table S3) (blue circles; gene Eucgr.L01734 was excluded) across the 11 chromosomes, their eQTL associations (turquoise triangles represent eQTL peaks), and nine QTLs (Additional file 1: Table S5) for lignin content/composition (total lignin content, S content, G content and S/G ratio) and sugar release (glucose release, xylose release and glucose + xylose release) overlapping with the candidate genes or their eQTL peak positions. **b** Genomic information for the QTL position, for example bin 6_1, can be browsed directly from the interactive Circos plot using the JBrowse tool. The position of the eQTL peak bin is delimited in turquoise in the first track (top), while the position of the visualized area is highlighted in red. The three subsequent tracks represent gene annotation in the genomic region and RNA-seq coverage in xylem and young leaves. All the transcriptomes available in EucGenIE can be visualized as additional tracks using the JBrowse tool

To further prioritize the best candidates associated with variation in cell wall properties in *Eucalyptus* secondary xylem, we took advantage of qtlXplorer to perform a systems genetics analysis focusing on the eight LAC/PRX candidates highlighted by the co-expression analysis (subnetwork 2 genes in Additional file 1: Table S3). After selecting the eight LAC/PRX genes in the “Genes” panel, we used the “Get eQTLs” function (using data from the *E. urophylla* backcross, F1 Hybrid map), to retrieve 14 eQTLs mapped to 7 chromosomes (13 mapped in *trans* and one in *cis*) (Fig. 5a). These eQTLs spanned 73.2 Mb across the 7 chromosomes, including 4519 underlying genes, retrieved using the function “Get underlying genes”, that could be browsed directly via the interactive Circos plot through the JBrowse tool (Fig. 5b) and saved as a tab-delimited text file. In addition, the function “Get overlapping Trait QTLs” allowed the identification of nine phenotypic traits related to wood composition and processing, that overlapped with the candidate genes or their eQTL peak positions (Fig. 5a, Additional file 1: Table S5), supporting a role of these genes in wood formation.

**Fig. 6:**
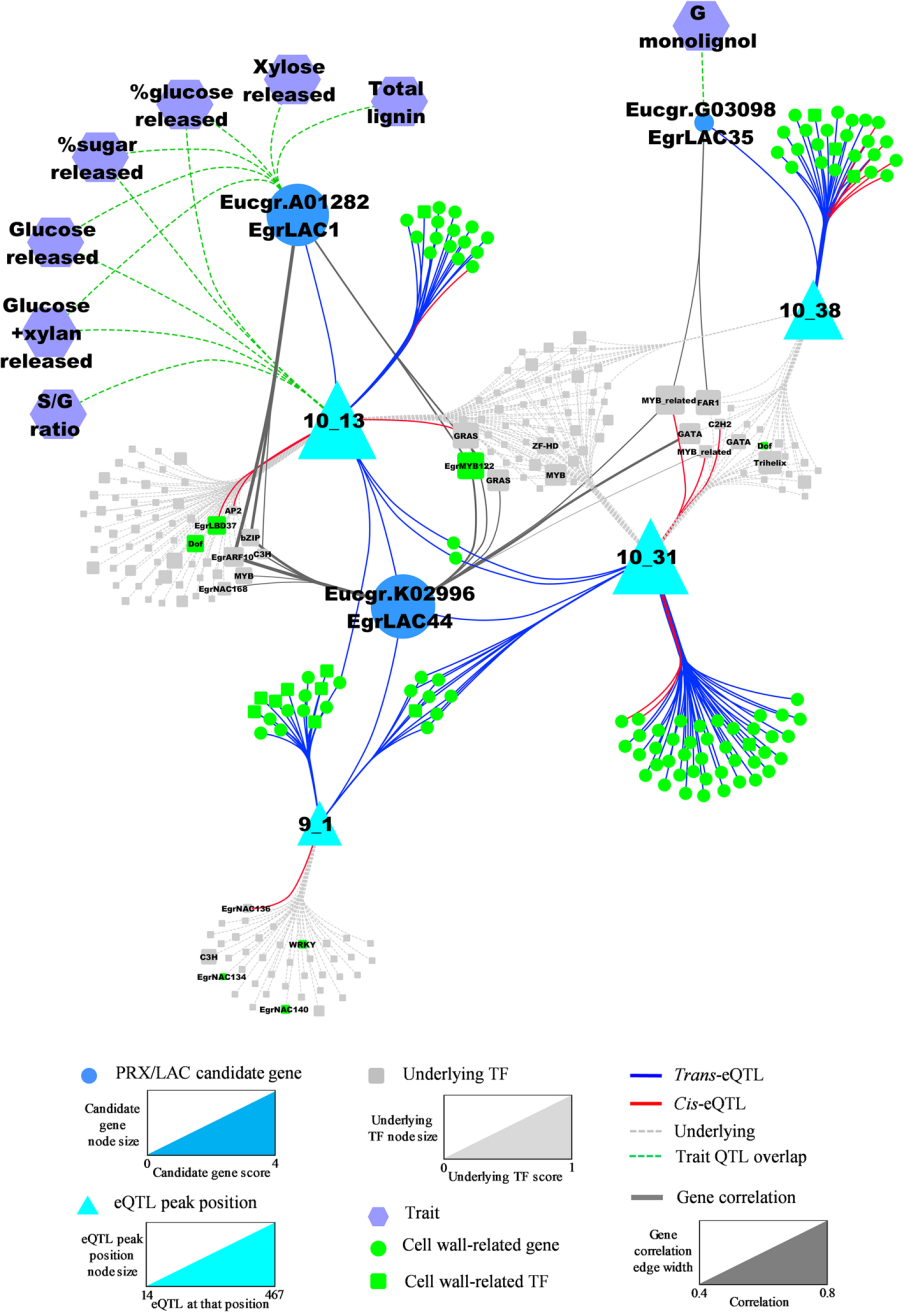
Systems genetics analysis of LAC/PRX genes associated with SCW biosynthesis in *Eucalyptus* xylem formation. The network was built from data exported from qtlXplorer and illustrates a systems genetics model for three candidate genes out of the eight previously presented. *Cis-* and *trans*-eQTL associations connecting candidate genes (blue nodes) or cell wall-related genes (green nodes) to eQTL peak positions (turquoise triangles) are represented by red and blue edges, respectively. Transcription factors (TFs) underlying eQTL peak positions are represented by grey or green (SCW-related) squares and connected to eQTL peak positions via grey dashed line edges. Gene expression profile correlations are represented by grey solid edges, with thickness proportional to the absolute value of the correlation. Physical overlap of trait QTLs (purple nodes) with candidate gene or eQTL peak positions are represented by green dashed lines. eQTL peak position node size is proportional to the number of genes having eQTLs (*cis* or *trans*) mapped at that genomic position. Underlying TF node size is proportional to the number and average value of the correlations of TFs with genes having *cis-* or *trans*-eQTLs mapped at that position (underlying TF score; Additional file 1: Table S8). Candidate gene node size is proportional to its score for prioritization (see Additional file 2: Methods S5; Additional file 1: Table S9), taking into account (i) their correlations (number and average value) with SCW genes across the population-wide transcriptomic data, (ii) the number of physical overlaps with candidate gene/SCW-related QTLs, (iii) the number of eQTLs mapped at SCW-enriched eQTL positions, (iv) the number of overlaps of their eQTL positions with SCW-related trait QTLs, and (v) the number of SCW-related TFs in the top 10 best candidate TFs underlying their eQTL positions

In order to exploit all the ‘omics data that can be mined through qtlXplorer and demonstrate the full power of this approach, we further visualized and analysed the final systems genetics model as a network using an external tool (Cytoscape) [67]. In this model (Fig. 6), nodes represent either genes (blue, green or grey), eQTL peak positions (turquoise) or phenotypic traits (purple), while edges represent different types of associations: *cis*- or *trans*-eQTL (red or blue arrows respectively), gene correlations (grey solid lines), overlap with eQTL peak positions (grey dashed lines), or overlap with trait QTLs (green dashed lines). Using the function “Get overlapping eQTLs” in qtlXplorer, we extracted a total of 1729 genes with 1821 eQTL peaks that co-localize with the eQTL peak positions of our eight candidates (Additional file 1: Table S6). These genes included 124 SCW genes with eQTLs mapped mainly to four positions (annotated 9_1, 10_13, 10_31 and 10_38), together with the eQTLs of three of the eight candidate genes (Eucgr.K02996/EgrLAC44, Eucgr.A01282/EgrLAC1, Eucgr.G03098/EgrLAC35; Additional file 1: Table S6, Table S7). Figure 6 gives the network around these three candidate LAC genes and four SCW-enriched eQTL positions as a simplified example (the complete model for all eight LAC/PRX candidate genes can be viewed in Additional file 4: Figure S2). Noteworthy, two of these candidate genes (Eucgr.A01282/EgrLAC1, Eucgr.G03098/EgrLAC35) and one of their eQTL peak positions overlapped with a number of QTLs for sugar release and lignin content/composition traits in secondary xylem of *Eucalyptus* (Fig. 6). These results support the hypothesis that some of these genes play a role in SCW formation in *Eucalyptus*.

Considering that the eQTL positions represent putative polymorphic regulatory loci, this approach allows prioritizing a set of underlying genes possibly involved in the regulation of the candidate genes. The function “Get underlying genes” of qtlXplorer, allowed the retrieving of a list of underlying genes for each eQTL peak position, from which we retained TFs as expected regulators of target genes. Subsequently, co-expression associations between TFs underlying an eQTL peak position and the genes with eQTLs mapped at that particular position were retrieved using the “Filter gene–gene correlation” function from the “Genes” panel in qtlXplorer. The underlying TFs with the highest number and stronger correlations were identified as the most probable regulatory genes causal of these eQTLs (Additional file 2: Method S5, Additional file 1: Table S8). For the three overlapping SCW-enriched eQTL positions on chromosome 10 (10_13, 10_31 and 10_38), the closest homolog of the SCW regulator *AtMYB43* in *Arabidopsis* (*EgrMYB122)* [68], appeared as one of the top candidate regulators (Fig. 6). Furthermore, for the two eQTL positions 10_13 and 9_1, other known regulators of SCW formation and/or involved in vascular development such as *EgrLBD37* in *Eucalyptus* [69] and homologs of *AtVNI1*, *AtWRKY13* and *AtDof2.1* in *Arabidopsis* [70–72], were detected underlying these positions. These results demonstrate that in addition to sharing eQTLs with SCW genes and overlapping with important wood growth and composition trait QTLs, these candidate genes might be under the control of major regulatory loci controlling SCW formation in *Eucalyptus* xylem. When calculating a score taking into account all these lines of evidence (see Additional file 2: Method S5, Additional file 1: Table S9), *EgrLAC44*, *EgrLAC1* and *EgrLAC35* appeared as top-ranked candidates supporting their direct involvement in lignin biosynthesis during xylogenesis in *Eucalyptus*.

Overall, this case study illustrates how a complex systems genetics model can be built using qtlXplorer to analyse the genetic architecture and investigate the function of candidate genes. The results showed an overrepresentation of LAC genes as potential candidates for lignin biosynthesis, in agreement with a higher contribution of LAC genes to SCW lignification in *Arabidopsis* [61], while the role of class III PRX genes seems to be restricted to biogenesis of cell wall domains in a tissue and/or cell type specific manner [46, 73, 74]. In addition, our approach related a large set of 51 PRX and LAC genes to defense or more generally stress responses, suggesting that several members of these expanded families are likely to be key determinants of perennial plants adaptation to their environment. The EucGenIE environment now provides a powerful combination of genomics and systems genetics tools to investigate the role of such multigenic families like Class III PRX and LAC in tree development and stress response.

### Future work

The successful integration of multi-dimensional plant ‘omics datasets into biologically meaningful and human-interpretable systems models will require advanced data science skills, including the application of next-generation artificial intelligence (AI) i.e. the integration of two or more dimensional ‘omics datasets [75, 76]. Towards this, a number of large scale plant genomics resources are currently available and new online ‘omics databases are being developed that will facilitate the transition to integrative AI approaches for systems modelling. We have developed qtlXplorer, a genetic variation module in PlantGenIE and EucGenIE for mining population genomics data. We will add other *Eucalyptus* population genetics datasets as well as datasets linked to other PlantGenIE subdomains in the future, for example the eQTL data from the poplar GWAS study in Mähler et al. [77].

A particular focus of PlantGenIE is to make available genomics data from forest tree species, which represent many key biological evolutionary innovations, such as secondary growth, stress resilience, adaptability allowing long life span, perenniality and dormancy. The EucGenIE online resource and qtlXplorer provide an intuitive environment to explore and integrate genomics, transcriptomics and systems genetics data with interactive search and visualization tools. Even though the EucGenIE database is focused on exploring *Eucalyptus* data, orthology inference methods allow switching between different PlantGenIE domains to explore the same gene list in other plant species. Therefore, PlantGenIE facilitates comparative genomic analyses in non-model species, building analyses on data available for well characterised species.

As demonstrated in the case study, incorporating different layers of ‘omics information in a systems genetics network-based model is a powerful approach to highlight key regulators responsible for co-regulation of genes and to provide links between genes and phenotypic traits. However, the manual integration of these networks and the analysis of complex systems models can be labour intensive. This raises the necessity of expanding the suite of population genomics tools in the future, by adding an eQTL network angling tool that would incorporate eQTL information with co-expression data and trait QTL overlap, to further facilitate systems genetics analyses. A new generation of integrative approaches based on machine learning methods could also represent a further evolution of these tools to fully explore the associations between genotype and phenotype, while keeping in mind the computational challenges associated with this type of integrative analysis [78].

We anticipate that the range of species available in PlantGenIE.org will expand as adequate data resources are developed and we would welcome involvement from the communities of researchers working on other plant species. Since EucGenIE is implemented using an open source generic system (GenIE-Sys), users wanting to integrate their own published data or develop and implement additional features can do so, or collaborate with the authors. We recommend cloning the current version of the site from GitHub as a starting point. EucGenIE provides standardized query and visualization tools to analyse experiments individually (e.g. exPlot, ExImage, exHeatmap) which is valuable to further explore previously published datasets. Furthermore, integration of larger numbers of diverse *Eucalyptus* transcriptome datasets into meta-gene expression networks (e.g. exNet) will allow greater resolution for identifying key role players (regulators) underlying the unique biology, adaptations and perennial lifestyles of eucalypt species.

## Conclusions

qtlXplorer, within the PlantGenIE.org environment, provides an intuitive platform for exploring and integrating tree genomics, transcriptomics and systems genetics data with interactive visualization tools.

## Supplementary Information

**Additional file 1: Table S1**. Metadata for RNA-seq datasets integrated in EucGenIE. In total 355 transcriptome datasets were integrated: 42 exAtlas datasets, 30 biotic interactions datasets and 283 transcriptomes from different F2 backcross individuals. **Table S2**. The 44 laccase genes detected in Eucalyptus with their corresponding BLASTP, HMMER and SignalP results. Genes encoding proteins with the three truncated domains of a canonical LAC and predicted to have a signal peptide were considered for analysis in this study. **Table S3**. Expression values in transcripts per million (TPM) of the 59 LAC/PRX genes, in the E. grandis exAtlas [35] and across biotic stress conditions [36–38]. **Table S4**. Secondary cell wall-related genes that were used for co-expression analysis. **Table S5**. Nine trait QTLs that co-locate with the eight LAC/PRX candidate genes from subnetwork 2 (Fig. 2, Table S3) or their eQTL positions. **Table S6**. Genes sharing eQTLs at the eleven eQTL positions associated with the eight LAC/PRX candidate genes from subnetwork 2 (Fig. 2, Additional file 1: Table S3). **Table S7**. Fisher's Exact Test results for enrichment of cell wall-related genes with eQTLs located at each of eleven eQTL positions, respectively, associated with the eight LAC/PRX candidate genes from subnetwork 2 (Fig. 2, Additional file 1: Table S3). **Table S8**. Transcription factors underlying the eleven eQTL peak positions associated with the eight LAC/PRX candidate genes from subnetwork 2 (Fig. 2, Additional file 1: Table S3). **Table S9**. Final score for ranking the eight LAC/PRX candidate genes from subnetwork 2 (Fig. 2, Additional file 1: Table S3) using multiple lines of evidence.

**Additional file 2: Method S1**. Expression profiling of RNA-seq datasets. **Method S2**. QTL and eQTL analysis of data in qtlXplorer. **Method S3**. Overview of the EucGenIE tools. **Method S4**. Translating genes between species within PlantGenIE. **Method S5**. Case study: Laccases and peroxidases.

**Additional file 3: Figure S1**. Co-expression analysis of 137 LAC/PRX genes using exNet in EucGenIE. (a) Correlation network representing 62 correlations between 42 (out of a total of 137) LAC/PRX genes, filtered at threshold 5. (b) Correlation network representing 1877 correlations between 90 LAC/PRX and 1043 other correlated genes, obtained after expanding at threshold 6. Correlation networks are based on 72 transcriptomic datasets (all exAtlas and biotic interactions datasets; sample collections 2–4 in Table 1) in EucGenIE and were visualized using the exNet tool.

**Additional file 4: Figure S2**. Systems genetics analysis of the eight LAC/PRX genes associated with secondary cell wall (SCW) biosynthesis in *Eucalyptus* xylem formation. The network was built from data exported from qtlXplorer and follows the same structure as presented in Fig. 6. *Cis-* and *trans*-eQTL associations connecting candidate genes (blue nodes) or cell wall-related genes (green nodes) to eQTL peak positions (turquoise triangles) are represented by red and blue edges, respectively. Transcription factors (TFs) underlying eQTL peak positions are represented by grey or green squares and connected to eQTL peak positions via grey dashed line edges. Gene expression profile correlations are represented by grey solid edges, with thickness proportional to the absolute value of the correlation. Physical overlap of trait QTLs (purple nodes) with candidate gene or eQTL peak positions are represented by green dashed lines. eQTL peak position node size is proportional to the number of genes having eQTLs (*cis* or *trans*) mapped at that genomic position. Underlying TF node size is proportional to the number and average value of the correlations of TFs with genes having *cis-* or *trans*-eQTLs mapped at that position (underlying TF score; Additional file 1: Table S8). Candidate gene node size is proportional to its score for prioritization (see Additional file 2: Methods S5; Additional file 1: Table S9), taking into account (i) their correlations (number and average value) with SCW genes across the population-wide transcriptomic data, (ii) the number of physical overlaps with candidate gene/SCW-related QTLs, (iii) the number of eQTLs mapped at SCW-enriched eQTL positions, (iv) the number of overlaps of their eQTL positions with SCW-related trait QTLs, and (v) the number of SCW-related TFs in the top 10 best candidate TFs underlying their eQTL positions.

## Data Availability

The datasets analysed during the current study can be explored via the web-based EucGenIE (https://eucgenie.org) resource within the PlantGenIE (https://plantgenie.org) platform. After RNA sequencing of wood forming tissues of 283 F2 backcross individuals, the population-wide RNA sequences have been deposited in the NCBI Sequence Read Archive, accession no. SUB2087452 (*E. urophylla* backcross population) and SUB4571814 (*E. grandis* backcross population). All other RNAseq datasets in EucGenIE have been published and data availability is described in those publications (see Additional file 1: Table S1).

## Abbreviations

AI: Artificial intelligence
AtGenIE: *Arabidopsis* Genome integrative explorer
ComPlEx: Comparative co-expression network conservation
ConGenIE: Conifer genome integrative explorer
eFP: Electronic fluorescent pictograph
eQTL: Expression quantitative trait loci
EucGenIE: *Eucalyptus* Genome integrative explorer
GenIE-Sys: Genome integrative explorer system
GWAS: Genome-wide association studies
LAC: Laccases
ME: Module eigengene
PlantGenIE: Plant genome integrative explorer
PopGenIE: Populus genome integrative explorer
PRX: Peroxidases
QTL: Quantitative trait loci
SCW: Secondary cell wall
ROS: Reactive oxygen species
TF: Transcription factor

## References

[1] Mackay TFC, Stone EA, Ayroles JF (2009). The genetics of quantitative traits: challenges and prospects. Nat Rev Genet.

[2] Mizrachi E, Myburg AA (2016). Systems genetics of wood formation. Curr Opin Plant Biol.

[3] Mizrachi E, Verbeke L, Christie N, Fierro AC, Mansfield SD, Davis MF (2017). Network-based integration of systems genetics data reveals pathways associated with lignocellulosic biomass accumulation and processing. Proc Natl Acad Sci.

[4] Misra BB, Langefeld C, Olivier M, Cox LA (2018). Integrated omics: tools, advances and future approaches. J Mol Endocrinol.

[5] Goodstein DM, Shu S, Howson R, Neupane R, Hayes RD, Fazo J (2012). Phytozome: a comparative platform for green plant genomics. Nucleic Acids Res.

[6] Bolser D, Staines D, Perry E, Kersey P (2017). Ensembl Plants: integrating tools for visualizing, mining, and analyzing plant genomic data. Methods Mol Biol.

[7] Gupta P, Naithani S, Tello-Ruiz MK, Chougule K, D’Eustachio P, Fabregat A (2016). Gramene database: navigating plant comparative genomics resources. Curr Plant Biol.

[8] Van Bel M, Diels T, Vancaester E, Kreft L, Botzki A, Van De Peer Y (2018). PLAZA 4.0: an integrative resource for functional, evolutionary and comparative plant genomics. Nucleic Acids Res.

[9] Berardini TZ, Reiser L, Li D, Mezheritsky Y, Muller R, Strait E (2015). The *Arabidopsis* information resource: making and mining the “gold standard” annotated reference plant genome. Genesis.

[10] Andorf CM, Cannon EK, Portwood JL, Gardiner JM, Harper LC, Schaeffer ML (2016). MaizeGDB update: new tools, data and interface for the maize model organism database. Nucleic Acids Res.

[11] Waese J, Provart NJ (2016). The bio-analytic resource: data visualization and analytic tools for multiple levels of plant biology. Curr Plant Biol.

[12] Sundell D, Mannapperuma C, Netotea S, Delhomme N, Lin YC, Sjödin A, et al. The plant genome integrative explorer resource: PlantGenIE.org. New Phytol. 2015;208(4):1149–56. 10.1111/nph.13557 26192091

[13] Winter D, Vinegar B, Nahal H, Ammar R, Wilson GV, Provart NJ (2007). An “electronic fluorescent pictograph” browser for exploring and analyzing large-scale biological data sets. PLoS ONE.

[14] Toufighi K, Brady SM, Austin R, Ly E, Provart NJ (2005). The botany array resource: e-northerns, expression angling, and promoter analyses. Plant J.

[15] Hruz T, Laule O, Szabo G, Wessendorp F, Bleuler S, Oertle L (2008). Genevestigator V3: a reference expression catabase for the meta-analysis of transcriptomes. Adv Bioinformatics.

[16] Mutwil M, Klie S, Tohge T, Giorgi FM, Wilkins O, Campbell MM (2011). PlaNet: combined sequence and expression comparisons across plant networks derived from seven species. Plant Cell.

[17] Gielen D, Boshell F, Saygin D, Bazilian MD, Wagner N, Gorini R (2019). The role of renewable energy in the global energy transformation. Energy Strateg Rev.

[18] Bastin JF, Finegold Y, Garcia C, Mollicone D, Rezende M, Routh D (2019). The global tree restoration potential. Science (80-).

[19] De La Torre AR, Birol I, Bousquet J, Ingvarsson PK, Jansson S, Jones SJM (2014). Insights into conifer giga-genomes. Plant Physiol.

[20] Wegrzyn JL, Staton MA, Street NR, Main D, Grau E, Herndon N (2019). Cyberinfrastructure to improve forest health and productivity: the role of tree databases in connecting genomes, phenomes, and the environment. Front Plant Sci.

[21] Falk T, Herndon N, Grau E, Buehler S, Richter P, Zaman S (2018). Growing and cultivating the forest genomics database. TreeGenes Database.

[22] Kremer A, Abbott AG, Carlson JE, Manos PS, Plomion C, Sisco P (2012). Genomics of Fagaceae. Tree Genet Genomes.

[23] Jung S, Ficklin SP, Lee T, Cheng CH, Blenda A, Zheng P (2014). The genome database for rosaceae (GDR): year 10 update. Nucleic Acids Res.

[24] Ficklin SP, Feltus FA (2011). Gene coexpression network alignment and conservation of gene modules between two grass species: maize and rice. Plant Physiol.

[25] Mannapperuma C, Waterworth J, Street N. GenIE-Sys: Genome Integrative Explorer System. bioRxiv [Internet]. 2019;808881. 10.1101/808881v1

[26] Netotea S, Sundell D, Street NR, Hvidsten TR (2014). ComPlEx: conservation and divergence of co-expression networks in *A. thaliana*, Populus and *O. sativa*. BMC Genomics.

[27] Sundell D, Street NR, Kumar M, Mellerowicz EJ, Kucukoglu M, Johnsson C (2017). AspWood: high-spatial-resolution transcriptome profiles reveal uncharacterized modularity of wood formation in *Populus tremula*. Plant Cell.

[28] Jokipii-Lukkari S, Sundell D, Nilsson O, Hvidsten TR, Street NR, Tuominen H (2017). NorWood: a gene expression resource for evo-devo studies of conifer wood development. New Phytol.

[29] Myburg AA, Grattapaglia D, Tuskan GA, Hellsten U, Hayes RD, Grimwood J (2014). The genome of *Eucalyptus grandis*. Nature.

[30] Strauss SH, Myburg AA (2015). Plant scientists celebrate new woody plant genome. New Phytol.

[31] Silva-junior OB, Faria DA, Grattapaglia D (2015). A flexible multi-species genome-wide 60K SNP chip developed from pooled resequencing of 240 *Eucalyptus* tree genomes across 12 species. New Phytol.

[32] Hefer CA, Mizrachi E, Joubert F, Myburg AA (2011). The *Eucalyptus* genome integrative explorer (EucGenIE): a resource for *Eucalyptus* genomics and transcriptomics. BMC Proc.

[33] Mizrachi E, Hefer CA, Ranik M, Joubert F, Myburg AA (2010). De novo assembled expressed gene catalog of a fast-growing *Eucalyptus* tree produced by Illumina mRNA-Seq. BMC Genomics.

[34] Mizrachi E, Maloney VJ, Silberbauer J, Hefer CA, Berger DK, Mansfield SD (2015). Investigating the molecular underpinnings underlying morphology and changes in carbon partitioning during tension wood formation in *Eucalyptus*. New Phytol.

[35] Vining K, Romanel E, Jones R (2014). The floral transcriptome of *Eucalyptus grandis*. New Phytol.

[36] Oates CN, Külheim C, Myburg AA, Slippers B, Naidoo S (2015). The transcriptome and terpene profile of *Eucalyptus grandis* reveals mechanisms of defense against the insect pest, *Leptocybe invasa*. Plant Cell Physiol.

[37] Mangwanda R, Myburg AA, Naidoo S (2015). Transcriptome and hormone profiling reveals Eucalyptus grandis defence responses against *Chrysoporthe austroafricana*. BMC Genomics.

[38] Meyer FE, Shuey LS, Naidoo S, Mamni T, Berger DK, Myburg AA (2016). Dual RNA-sequencing of *Eucalyptus nitens* during *Phytophthora cinnamomi* challenge reveals pathogen and host factors influencing compatibility. Front Plant Sci.

[39] Christie N, Myburg AA, Joubert F, Murray SL, Carstens M, Lin Y (2017). Systems genetics reveals a transcriptional network associated with susceptibility in the maize – grey leaf spot pathosystem. Plant J.

[40] Krzywinski M, Schein J, Birol I, Connors J, Gascoyne R, Horsman D (2009). Circos: an information aesthetic for comparative genomics. Genome Res.

[41] Hanson R, Tacy A. GWT in Action: Easy Ajax with the Google Web Toolkit. Manning Publications Co.; 2007.

[42] Liu Q, Luo L, Zheng L (2018). Lignins: biosynthesis and biological functions in plants. Int J Mol Sci.

[43] Chanoca A, de Vries L, Boerjan W (2019). Lignin engineering in forest trees. Front Plant Sci.

[44] Berthet S, Thevenin J, Baratiny D, Demont-Caulet N, Debeaujon I, Bidzinski P (2012). Role of plant laccases in lignin polymerization. Adv Bot Res.

[45] Barros J, Serk H, Granlund I, Pesquet E (2015). The cell biology of lignification in higher plants. Ann Bot.

[46] Francoz E, Ranocha P, Nguyen-Kim H, Jamet E, Burlat V, Dunand C (2015). Roles of cell wall peroxidases in plant development. Phytochemistry.

[47] Tobimatsu Y, Schuetz M (2019). Lignin polymerization: how do plants manage the chemistry so well?. Curr Opin Biotechnol.

[48] Wang J, Feng J, Jia W, Chang S, Li S, Li Y (2015). Lignin engineering through laccase modification: a promising field for energy plant improvement. Biotechnol Biofuels.

[49] Shigeto J, Tsutsumi Y (2016). Diverse functions and reactions of class III peroxidases. New Phytol.

[50] Almagro L, Gómez Ros LV, Belchi-Navarro S, Bru R, Ros Barceló A, Pedreño MA (2009). Class III peroxidases in plant defence reactions. J Exp Bot.

[51] Liang M, Haroldsen V, Cai X, Wu Y (2006). Expression of a putative laccase gene, ZmLAC1, in maize primary roots under stress. Plant, Cell Environ.

[52] Arcuri MLC, Fialho LC, Vasconcellos Nunes-Laitz A, Fuchs-Ferraz MCP, Wolf IR, Valente GT (2020). Genome-wide identification of multifunctional laccase gene family in *Eucalyptus grandis*: potential targets for lignin engineering and stress tolerance. Trees Struct Funct.

[53] Li Q, Yu H, Cao PB, Fawal N, Mathé C, Azar S (2015). Explosive tandem and segmental duplications of multigenic families in *Eucalyptus grandis*. Genome Biol Evol.

[54] Carocha V, Soler M, Hefer C, Cassan-Wang H, Fevereiro P, Myburg AA (2015). Genome-wide analysis of the lignin toolbox of *Eucalyptus grandis*. New Phytol.

[55] Goicoechea M, Lacombe E, Legay S, Mihaljevic S, Rech P, Jauneau A (2005). EgMYB2, a new transcriptional activator from *Eucalyptus* xylem, regulates secondary cell wall formation and lignin biosynthesis. Plant J.

[56] Ployet R, Veneziano Labate MT, Regiani Cataldi T, Christina M, Morel M, San Clemente H (2019). A systems biology view of wood formation in *Eucalyptus grandis* trees submitted to different potassium and water regimes. New Phytol.

[57] Hussey SG, Mizrachi E, Creux NM, Myburg AA (2013). Navigating the transcriptional roadmap regulating plant secondary cell wall deposition. Front Plant Sci.

[58] Zhong R, Ye Z-H (2012). MYB46 and MYB83 bind to the SMRE sites and directly activate a suite of transcription factors and secondary wall biosynthetic genes. Plant Cell Physiol.

[59] Zhong R, Lee C, Ye Z-H (2010). Global analysis of direct targets of secondary wall NAC master switches in *Arabidopsis*. Mol Plant.

[60] Berthet S, Demont-Caulet N, Pollet B, Bidzinski P, Cézard L, Le Bris P (2011). Disruption of LACCASE4 and 17 results in tissue-specific alterations to lignification of *Arabidopsis thaliana* stems. Plant Cell.

[61] Zhao Q, Nakashima J, Chen F, Yin Y, Fu C, Yun J (2013). Laccase is necessary and nonredundant with peroxidase for lignin polymerization during vascular development in *Arabidopsis*. Plant Cell.

[62] Lee Y, Rubio MC, Alassimone J, Geldner N (2013). A mechanism for localized lignin deposition in the endodermis. Cell.

[63] Lu S, Li Q, Wei H, Chang M-J, Tunlaya-Anukit S, Kim H (2013). Ptr-miR397a is a negative regulator of laccase genes affecting lignin content in *Populus trichocarpa*. Proc Natl Acad Sci U S A.

[64] Chen H, Wang JP, Liu H, Li H, Lin Y-CJ, Shi R, et al. Hierarchical transcription-factor and chromatin binding network for wood formation in *Populus trichocarpa*. Plant Cell. 2019;31:602–26. 10.1105/tpc.18.00620 PMC6482634 30755461

[65] Ranocha P, Chabannes M, Chamayou S, Jauneau A, Boudet A-M, Goffner D (2002). Laccase down-regulation causes alterations in phenolic metabolism and cell wall structure in poplar. Plant Physiol.

[66] Bryan AC, Jawdy S, Gunter L, Gjersing E, Sykes R, Hinchee MAW (2016). Knockdown of a laccase in *Populus deltoides* confers altered cell wall chemistry and increased sugar release. Plant Biotechnol J.

[67] Smoot ME, Ono K, Ruscheinski J, Wang P-L, Ideker T (2011). Cytoscape 2.8: new features for data integration and network visualization. Bioinformatics.

[68] Zhong R, Lee C, Zhou J, McCarthy RL, Ye Z-H (2008). A battery of transcription factors involved in the regulation of secondary cell wall biosynthesis in Arabidopsis. Plant Cell.

[69] Lu Q, Shao F, Macmillan C, Wilson IW, van der Merwe K, Hussey SG (2018). Genomewide analysis of the lateral organ boundaries domain gene family in Eucalyptus grandis reveals members that differentially impact secondary growth. Plant Biotechnol J.

[70] Yamaguchi M, Goué N, Igarashi H, Ohtani M, Nakano Y, Mortimer JC (2010). VASCULAR-RELATED NAC-DOMAIN6 and VASCULAR-RELATED NAC-DOMAIN7 effectively induce transdifferentiation into xylem vessel elements under control of an induction system. Plant Physiol.

[71] Li W, Tian Z, Yu D (2015). WRKY13 acts in stem development in *Arabidopsis thaliana*. Plant Sci an Int J Exp plant Biol.

[72] Gardiner J, Sherr I, Scarpella E (2010). Expression of DOF genes identifies early stages of vascular development in *Arabidopsis* leaves. Int J Dev Biol.

[73] Yi Chou E, Schuetz M, Hoffmann N, Watanabe Y, Sibout R, Samuels AL (2018). Distribution, mobility, and anchoring of lignin-related oxidative enzymes in *Arabidopsis* secondary cell walls. J Exp Bot.

[74] Hoffmann N, Benske A, Betz H, Schuetz M, Lacey SA (2020). Laccases and peroxidases co-localize in lignified secondary cell walls throughout stem development. Plant Physiol.

[75] Barone L, Williams J, Micklos D (2017). Unmet needs for analyzing biological big data: a survey of 704 NSF principal investigators. PLoS Comput Biol.

[76] Harfouche AL, Jacobson DA, Kainer D, Romero JC, Harfouche AH, Scarascia Mugnozza G (2019). Accelerating climate resilient plant breeding by applying next-generation artificial intelligence. Trends Biotechnol.

[77] Mähler N, Wang J, Terebieniec BK, Ingvarsson PK, Street NR, Hvidsten TR (2017). Gene co-expression network connectivity is an important determinant of selective constraint. PLoS Genet.

[78] Mirza B, Wang W, Wang J, Choi H, Chung NC, Ping P (2019). Machine learning and integrative analysis of biomedical big data. Genes (Basel).

[79] Ward J (1963). Hierarchical grouping to optimize an objective function. J Am Stat Assoc.

