# Supplementary material for: qtlXplorer: an online systems genetics browser in the Eucalyptus Genome Integrative Explorer (EucGenIE)
Source: BMC Bioinformatics. 2021 Dec 15;22:595. doi: 10.1186/s12859-021-04514-9 (PMC8672637; doi:10.1186/s12859-021-04514-9)
Supplement: Supplementary file 2 — Additional file 2: Method S1. Expression profiling of RNA-seq datasets. Method S2. QTL and eQTL analysis of data in qtlXplorer. Method S3. Overview of the EucGenIE tools. Method S4. Translating genes between species within PlantGenIE. Method S5. Case study: Laccases and peroxidases. [file 12859_2021_4514_MOESM2_ESM.docx]

**qtlXplorer: An online systems genetics browser in the *Eucalyptus* Genome Integrative Explorer (EucGenIE)**

Nanette Christie^1‡*^, Chanaka Mannapperuma^2‡^, Raphael Ployet^1‡^, Karen van der Merwe^1^, Niklas Mähler^2^, Nicolas Delhomme^3^, Sanushka Naidoo^1^, Eshchar Mizrachi^1^, Nathaniel R. Street^2*^, Alexander A. Myburg^1^

^1^Department of Biochemistry, Genetics and Microbiology, Forestry and Agricultural Biotechnology Institute (FABI), University of Pretoria, Private bag X20, Pretoria, 0028, South Africa; ^2^Umeå Plant Science Centre, Department of Plant Physiology, Umeå University, SE-907 81 Umeå, Sweden; ^3^ Umeå Plant Science Centre, Department of Forest Genetics and Plant Physiology, Swedish University of Agricultural Sciences, SE-901 83 Umeå, Sweden.

‡ These authors contributed equally to this work

*Correspondence: Nanette Christie, E-mail: [nanette.christie@fabi.up.ac.za](mailto:zander.myburg@up.ac.za)

Nathaniel R. Street, E-mail: [nathaniel.street@umu.se](mailto:nathaniel.street@umu.se)

**Key words (3-10 keywords):** qtlXplorer, *Eucalyptus*, EucGenIE, systems genetics, eQTL, co-expression, ‘omics integration, online resource, database, genome browser

**Additional File 2: Method S1. Expression profiling of RNA-seq datasets**

Population RNA-seq datasets (to be explored with qtlXplorer)

RNA sequencing of wood forming tissues of 283 F2 backcross individuals (156 from the *E. urophylla* and 118 from the *E. grandis* backcross population, respectively) was performed. The population-wide sequences have been deposited in the NCBI Sequence Read Archive, accession no. SUB2087452 (*E. urophylla* backcross population) and SUB4571814 (*E. grandis* backcross population). Fragments Per Kilobase of transcript per Million mapped reads (FPKM) were calculated per gene model using TopHat version 1.3 and Cufflinks version 1.0.3 (bias correction and quartile normalization was enabled for the FPKM calculation) [1,2] for each backcross population. The R package WGCNA v1.62 [3] was used for generating modules of highly correlated genes based on the gene expression profiles across individuals, for each backcross population. In order to ensure good scale free topology fit of the networks (R^2^ ≥ 0.8), soft thresholding powers of 4 and 2, were used to calculate adjacency matrices for the *E. urophylla* and *E. grandis* backcross populations respectively.

RNA-seq datasets (to be explored with Expression tools)

All RNA-seq datasets were pre-processed using the pipeline detailed in Delhomme *et al.* [4]. In summary, raw data quality was assessed using FastQC (http://www.bioinformatics.babraham.ac.uk/projects/fastqc/) and residual ribosomal RNA (rRNA) sequences were removed using SortMeRNA v.2.1 [5] (settings: –log –paired_in –fastx–sam –num_alignments 1).  Data was then filtered to remove adapters and trimmed for quality using Trimmomatic v.0.32 [6] (settings: TruSeq3-PE-2.fa:2:30:10 LEADING:3 SLIDINGWINDOW:5:20 MINLEN:50). After completing the above steps, FastQC was run again to ensure the quality and make sure that no technical artefacts had been introduced. Filtered and pre-processed data were aligned to version 2 of the *E. grandis* reference genome using STAR v.2.5.2b [7] (nondefault settings: –outSAMstrandField intronMotif –readFilesComman d zcat–outSAMmapqUnique 254 –quantMode TranscriptomeSAM –outFilterMultimapNmax 100 –outRe adsUnmapped Fastx –chimSegmentMin 1 –outSAMtype BAM SortedByCoordinate–outWigType bedGraph –alignIntronMax 11000) and Kallisto [8]. The number of reads aligning to annotated gene models was determined using HTSEQ [9] for STAR output. These read counts were normalised using variance stabilizing transformation (VST; using blind = TRUE) implemented in the R-package DESeq2 v.1.13.14 [10]. Kallisto v0.43.1 was used to generate Transcripts Per Million (TPM) values. Finally, TPM and VST expression values were loaded into a MySQL database. VST normalized values were used to construct gene co-expression networks and TPM values were used in the expression visualization tools. The use of TPM values enables intuitive comparison of expression among different experiments by correcting for sequencing depth.

**Additional File 2: Method S2. QTL and eQTL analysis of data in qtlXplorer**

Complex trait analysis

Diameter (cm) at breast height (DBH) of the main stem was assessed as described previously [11]. Bark thickness was calculated as the difference between over-bark and under-bark DBH measurements. A wood disk taken at breast height (1.35 m) was used to determine wood basic density using the water displacement method (www.tappi.org/content/SARG/T258.pdf). Chemical wood properties were assessed using different analytical methods, including pyrolysis molecular beam mass spectrometry (pyMBMS) using previously published methodology [12]. HPLC analysis of cell wall sugars and Klason lignin composition analysis were performed according to Maloney and Mansfield [13].

Trait QTL and eQTL mapping

Four framework genetic linkage maps consisting of 130 (*E. urophylla* backcross, F1 Hybrid map), 112 (*E. urophylla* backcross, backcross parent map), 159 (*E. grandis* backcross, F1 Hybrid map) and 100 (*E. grandis* backcross, backcross parent map) Diversity arrays technology (DArT) markers were previously constructed for this F2 pseudo-backcross mapping pedigree of *E. grandis × E. urophylla* [14]. QTL and eQTL mapping was conducted, per map, using QTL Cartographer [15]. A walking speed of 1 cM was used in composite interval mapping with forward regression and backward elimination (P ≤ 0.1). Permutation-based likelihood ratio score thresholds were calculated to globally approximate α ≤ 0.05 experiment-wise [16].

eQTL classification and hotspot identification

eQTL that were located further than half the average size of an eQTL from the location of its linked gene, often on a different chromosome, were classified as *trans*-eQTL. Global *trans*-eQTL hotspots were identified by firstly calculating the genome-wide frequency of eQTLs and then normalising for local gene density, using the eQTL data analysis pipeline described in Christie *et al.* [17].

Trait correlation and QTL overlap analyses

To relate gene expression and trait profiles, all-versus-all gene-gene, gene-trait and trait-trait Pearson correlations were pre-calculated and stored in the EucGenIE database. Furthermore, all-versus-all eQTL-eQTL, eQTL-trait QTL and trait QTL-trait QTL overlap analyses were performed. A hybrid QTL overlap score was calculated for each pair of QTL, as detailed in Mizrachi *et al.* [18]. The score ranges from 0 (indicating no overlap) to 1 (indicating identical peak positions and perfect overlap).

**Additional File 2: Method S3. Overview of the EucGenIE tools**

The GeneList tool is the central tool (**Table 1**), where users can search and filter for genes based on different types of annotation and subsequently save custom gene lists. It is also possible to populate cross-species gene lists via the cross-species GeneList tool. The Cross-species GeneList uses the ortholog translation tables to translate gene lists between the species available in PlantGenIE; the user can use best DIAMOND hits (or reciprocal best DIAMOND hits) or the PLAZA resource ortholog methods described in **Additional File 2: Method S2** [19]. After selecting a starting gene list, other EucGenIE tools can be used to explore the data and refine the active gene list for further exploration.

Standard analysis features are provided in Genome tools and Analysis tools (**Table 1**). The SeqSearch tool extracts coding, transcript, protein or genomic sequences from the EucGenIE database. The BLAST tool compares input sequences to the EucGenIE sequence database to identify homologous sequence matches. The ChrDiagram tool plots the location of genes in the active gene list on the 11 chromosomes. The JBrowse tool is a genome browser allowing the genomic visualization of various data types. Enrichment is a tool that calculates enrichment for Gene Ontology (GO) categories, Kyoto Encyclopedia of Genes and Genomes (KEGG) pathways and Protein family (Pfam) domains in the active gene list. For the Enrichment tool, a GO overrepresentation approach that examines each term in the context of its parent terms was incorporated (the parent–child approach) [20]. The Venn tool generates an interactive Venn diagram for the saved gene lists in EucGenIE, and intersecting lists can be saved as new gene lists. The Venn tool is based on the interactive Venn diagram viewer (jvenn) [21].

exNet visualizes co-expression between genes in the active gene list, across the experiments in sample collections 2, 3 and 4 (**Additional File 1: Table S1**). A co-expression network is constructed where nodes represent genes and edges represent co-expression above a selected threshold. Increasing or decreasing the threshold leads to contraction or expansion of the network. Nodes (genes) are coloured according to the tissue with the highest expression in the *E. grandis* tissues exAtlas. At any point, the genes in a co-expression network can be saved as a new gene list. In exNet, networks are displayed using cytoscape.js [22] and gene co-expression networks are inferred using context likelihood of relatedness (CLR) values [23]. The remaining Expression tools allow data exploration within a selected sample collection. exImage provides a pictorial representation of the expression levels of a single gene (in the active gene list) across different tissues or conditions in the sample collection. exMatch finds genes with a chosen expression profile, using a color legend. A user can create the desired pictorial representation of expression levels and search for genes with this expression profile. exPlot visualizes expression profiles of the genes in the active gene list as line graphs across tissues or conditions in the sample collection. exHeatmap generates a heatmap plot of the expression levels across tissues or conditions, from where clusters of similarly expressed genes can interactively be selected and saved as new gene lists. exHeatmap is based on the Interactive Cluster Heatmap library (InChlib) [24].

**Additional File 2: Method S4. Translating genes between species within PlantGenIE**

The "translation" database contains all the ortholog information of species included in PlantGenIE, to enable users to move between different tools and subdomains. It contains data from the best DIAMOND hits (and reciprocal best DIAMOND hits) [25], as well as a combination of four methods as used and implemented in the PLAZA resource [19]: (i) Orthologous gene families (ORTHO) inferred using OrthoMCL, *i.e.* sub-families; (ii) Tree-based orthologs (TROG) inferred using tree reconciliation of the phylogenetic tree of a gene family; (iii) Best-Hits-and-Inparalogs (BHIF) inferred from BLAST hits against the PLAZA protein database, *i.e.* the best BLAST hit including in-paralogs (genes from the same species having higher bit scores than the best hit from any other species); and (iv) Anchor points (APs) referring to gene-based collinearity between species (providing information about putative positional orthologs). The integration of PLAZA within PlantGenIE is an ongoing effort and the major focus of the next release of the resource.

**Additional File 2: Method S5. Case study: Laccases and peroxidases**

Identification of LAC candidate genes in *E. grandis*

Seventeen LAC genes were previously annotated in the *A. thaliana* genome [26]. Accessions of 84 putative LAC encoding genes were retrieved using the GeneList tool in EucGenIE, using the keyword “laccase” as query. Among these, 76 genes with an average sequence identity greater than or equal to 40% to a typical LAC gene in *Arabidopsis* (AtLAC4; AT2G38080.1) were retained when analysed using the BLASTP tool in EucGenIE. Their sequences were retrieved using SeqSearch in EucGenIE and screened for the presence and integrity of the three PFAM domains (PF00394, PF07731, PF07732) via the online interface for biosequence analysis using profile hidden Markov Models (HMMER;<https://www.ebi.ac.uk/Tools/hmmer/>) [27]. The presence of a signal peptide was predicted using the SignalP 5.0 server (<http://www.cbs.dtu.dk/services/SignalP/>) [28]. Genes encoding proteins with the three untruncated domains of a canonical LAC protein and predicted to have a signal peptide, were considered as LAC genes for later analyses (**Additional File 1: Table S2**). This included 34 genes recently annotated as LAC genes in *E. grandis* by Arcuri *et al*. [29].

Systems genetics integration

For the systems genetics analysis of the eight PRX/LAC genes selected in the co-expression analysis, we extracted (i) eQTL associations, (ii) genes underlying eQTLs, (iii) overlapping trait QTLs, and (iv) gene correlations, from the *E. urophylla* F1 hybrid backcross population datasets using qtlXplorer. For this approach, we considered nine traits related to secondary cell wall properties and/or wood processing (listed in **Additional File 1: Table S5**), and for which trait QTL overlapped with the candidate genes or their eQTL positions. Candidate gene positions in the genome, their eQTL associations and overlaps with trait QTL were first visualized using the Circos tool in qtlXplorer, and then converted to a network using Cytoscape [30]. *Cis-* and *trans*-eQTL associations, physical overlap of eQTL positions or candidate genes with trait QTLs, physical overlap between eQTL position and TFs (underlying TFs), and candidate genes - underlying TFs correlations, were considered as edges in the network. For each eQTL position, the underlying TFs potentially responsible for the eQTLs were prioritized according to an underlying TF score: the number of correlations greater than 0.5 for each TF (with all genes sharing an eQTL at that locus) was calculated as a percentage of the maximum number of correlations possible for that eQTL multiplied by the average correlation (**Additional File 1: Table S8**). In the final model, the presence of SCW-related TFs ranked in the top 10 best underlying candidate TFs, was used to prioritize the PRX/LAC candidates most likely involved in SCW formation. For each of the eight PRX/LAC, a score was computed by taking into account (i) the number of correlations with SCW genes in the population-wide transcriptomic data, (ii) average absolute value of the correlations with SCW genes, (iii) number of physical overlaps with SCW-related QTLs, (iv) number of associations with SCW-enriched eQTLs, (v) number of overlaps with SCW-related QTLs of their eQTL positions, and (vi) number of SCW-related underlying TFs prioritized for the corresponding eQTL position (see **Additional File 1: Table S9** for the exact calculation). For the cell wall enrichment test, all genes annotated with the GO term “GO0071554 cell wall organization or biogenesis” or its child terms, were considered as cell wall-related. For each eQTL position, enrichment for cell wall-related genes with eQTLs mapped at that position was determined using a two sided Fisher’s Exact Test (p-value < 0.05; **Additional File 1: Table S7**).

**References:**

1. Kim D, Pertea G, Trapnell C, Pimentel H, Kelley R, Salzberg SL. TopHat2: Accurate alignment of transcriptomes in the presence of insertions, deletions and gene fusions. Genome Biol. 2013;14(4):1–13.

2. Trapnell C, Roberts A, Goff L, Pertea G, Kim D, R D, et al. Differential gene and transcript expression analysis of RNA-seq experiments with TopHat and Cufflinks. Nat Protoc. 2013;7(3):562–78.

3. Langfelder P, Zhang B, Horvath S. Defining clusters from a hierarchical cluster tree: the Dynamic Tree Cut package for R. Bioinformatics. 2008 Mar 1;24(5):719–20.

4. Delhomme N, Mähler N, Schiffthaler B, Sundell D. Guidelines for RNA-Seq data analysis. Epigenesys Protoc. 2014;

5. Kopylova E, Noé L, Touzet H. SortMeRNA: Fast and accurate filtering of ribosomal RNAs in metatranscriptomic data. Bioinformatics. 2012;28(24):3211–7.

6. Bolger AM, Lohse M, Usadel B. Trimmomatic: A flexible trimmer for Illumina sequence data. Bioinformatics. 2014;30(15):2114–20.

7. Dobin A, Davis CA, Schlesinger F, Drenkow J, Zaleski C, Jha S, et al. STAR: ultrafast universal RNA-seq aligner. Bioinformatics. 2013;29(1):15–21.

8. Bray NL, Pimentel H, Melsted P, Pachter L. Near-optimal probabilistic RNA-seq quantification. Nat Biotechnol. 2016;34(5):525–7.

9. Anders S, Pyl PT, Huber W. HTSeq-A Python framework to work with high-throughput sequencing data. Bioinformatics. 2015;31(2):166–9.

10. Love MI, Huber W, Anders S. Moderated estimation of fold change and dispersion for RNA-seq data with DESeq2. Genome Biol. 2014;15(12):550.

11. Kullan AR, van Dyk MM, Hefer CA, Jones N, Kanzler A, Myburg AA. Genetic dissection of growth, wood basic density and gene expression in interspecific backcrosses of *Eucalyptus grandis* and *E. urophylla*. BMC Genet. 2012;13:60.

12. Studer A, Zhao Q, Ross-Ibarra J, Doebley J. Identification of a functional transposon insertion in the maize domestication gene tb1. Nat Genet. 2011;43:1160–1163.

13. Maloney VJ, Mansfield SD. Characterization and varied expression of a membrane-bound endo-β-1,4-glucanase in hybrid poplar. Plant Biotechnol J. 2010;8(3):294–307.

14. Kullan ARK, van Dyk MM, Jones N, Kanzler A, Bayley A, Myburg AA. High-density genetic linkage maps with over 2,400 sequence-anchored DArT markers for genetic dissection in an F2 pseudo-backcross of *Eucalyptus grandis x E. urophylla*. Tree Genet Genomes. 2011;8:163–75.

15. Basten CJ, Weir BS, Zeng Z-B. QTL Cartographer, Version 1.16. 2002.

16. Doerge RW, Churchill GA. Permutation tests for multiple loci affecting a quantitative character. Genetics. 1996;142:285–94.

17. Christie N, Myburg AA, Joubert F, Murray SL, Carstens M, Lin Y, et al. Systems genetics reveals a transcriptional network associated with susceptibility in the maize – grey leaf spot pathosystem. Plant J. 2017;89:746–63.

18. Mizrachi E, Verbeke L, Christie N, Fierro AC, Mansfield SD, Davis MF. Network-based integration of systems genetics data reveals pathways associated with lignocellulosic biomass accumulation and processing. Proc Natl Acad Sci. 2017;114(5):1195–200.

19. Proost S, Bel M V, Vaneechoutte D, VanDePeer Y, Inzé D, Mueller-Roeber B, et al. PLAZA 3.0: An access point for plant comparative genomics. Nucleic Acids Res. 2015;43(D1):D974–81.

20. Grossmann S, Bauer S, Robinson PN, Vingron M. Improved detection of overrepresentation of Gene-Ontology annotations with parent-child analysis. Bioinformatics. 2007;23(22):3024–31.

21. Bardou P, Mariette J, Escudié F, Djemiel C, Klopp C. Jvenn: An interactive Venn diagram viewer. BMC Bioinformatics. 2014;15(1):1–7.

22. Franz M, Lopes CT, Huck G, Dong Y, Sumer O, Bader GD. Cytoscape.js: A graph theory library for visualisation and analysis. Bioinformatics. 2016;32(2):309–11.

23. Faith JJ, Hayete B, Thaden JT, Mogno I, Wierzbowski J, Cottarel G, et al. Large-scale mapping and validation of *Escherichia coli* transcriptional regulation from a compendium of expression profiles. PLoS Biol. 2007;5(1):0054–66.

24. Škuta C, Bartůněk P, Svozil D. Supplementary information InCHlib – Interactive Cluster Heatmap for web applications. J Cheminform. 2014;6(44):1–6.

25. Buchfink B, Xie C, Huson DH. Fast and sensitive protein alignment using DIAMOND. Nat Methods. 2015;12:59–60.

26. McCaig BC, Meagher RB, Dean JFD. Gene structure and molecular analysis of the laccase-like multicopper oxidase (LMCO) gene family in *Arabidopsis thaliana*. Planta. 2005;221(5):619–36.

27. Potter SC, Luciani A, Eddy SR, Park Y, Lopez R, Finn RD. HMMER web server: 2018 update. Nucleic Acids Res. 2018 Jul;46(W1):W200–4.

28. Almagro Armenteros JJ, Tsirigos KD, Sønderby CK, Petersen TN, Winther O, Brunak S, et al. SignalP 5.0 improves signal peptide predictions using deep neural networks. Nat Biotechnol. 2019 Apr;37(4):420–3.

29. Arcuri MLC, Fialho LC, Vasconcellos Nunes-Laitz A, Fuchs-Ferraz MCP, Wolf IR, Valente GT, et al. Genome-wide identification of multifunctional laccase gene family in *Eucalyptus grandis*: potential targets for lignin engineering and stress tolerance. Trees - Struct Funct. 2020;34(3):745–58.

30. Smoot ME, Ono K, Ruscheinski J, Wang P-L, Ideker T. Cytoscape 2.8: new features for data integration and network visualization. Bioinformatics. 2011;27(3):431–2.
