# Supplementary figures and images for: qtlXplorer: an online systems genetics browser in the Eucalyptus Genome Integrative Explorer (EucGenIE)
Source: BMC Bioinformatics. 2021 Dec 15;22:595. doi: 10.1186/s12859-021-04514-9 (PMC8672637; doi:10.1186/s12859-021-04514-9)

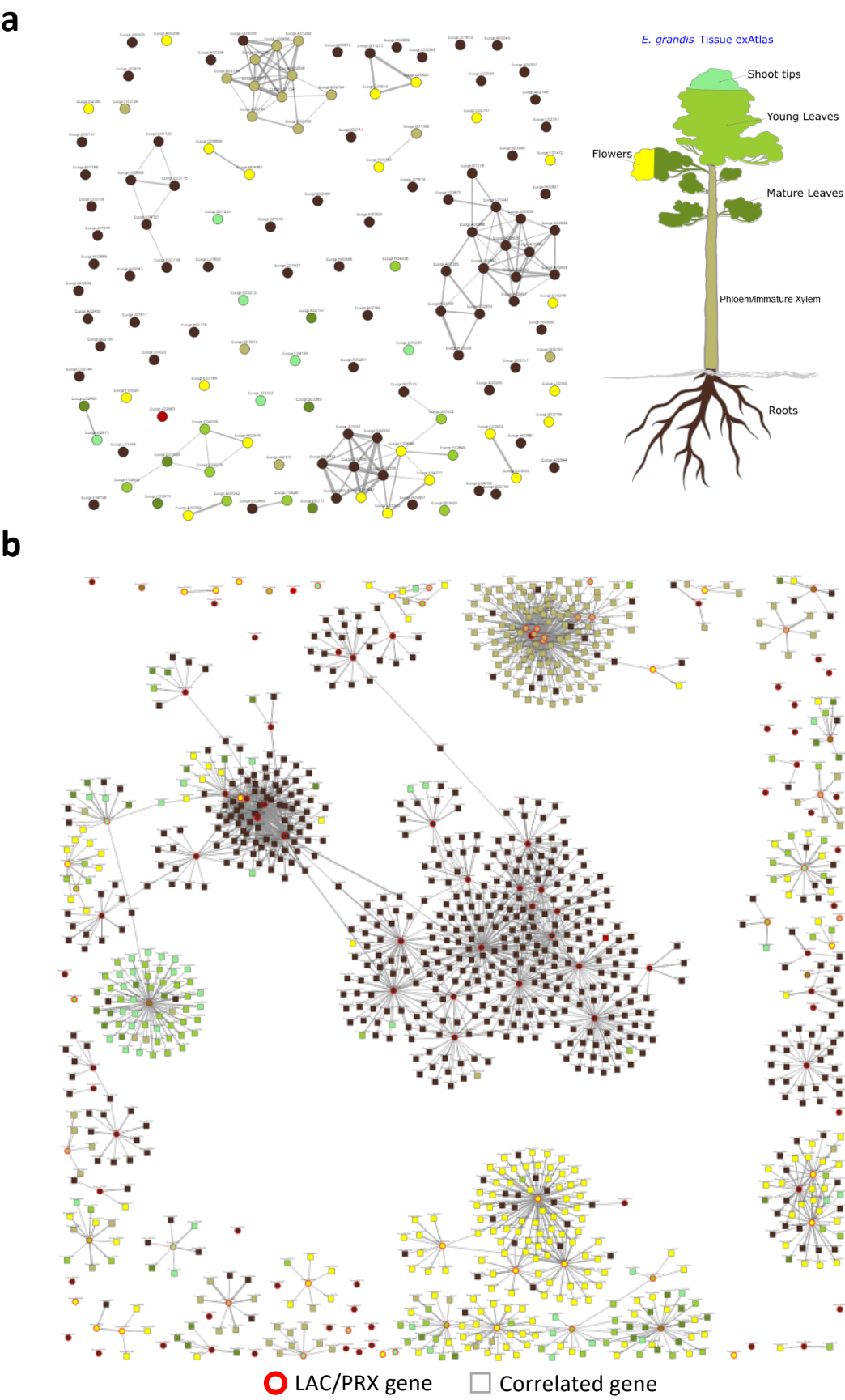

Supplement: Supplementary file 3 — Additional file 3: Figure S1. Co-expression analysis of 137 LAC/PRX genes using exNet in EucGenIE. (a) Correlation network representing 62 correlations between 42 (out of a total of 137) LAC/PRX genes, filtered at threshold 5. (b) Correlation network representing 1877 correlations between 90 LAC/PRX and 1043 other correlated genes, obtained after expanding at threshold 6. Correlation networks are based on 72 transcriptomic datasets (all exAtlas and biotic interactions datasets; sample collections 2–4 in Table 1) in EucGenIE and were visualized using the exNet tool. [file 12859_2021_4514_MOESM3_ESM.pdf]

Christie *et al.*, 2021  
Figure S2

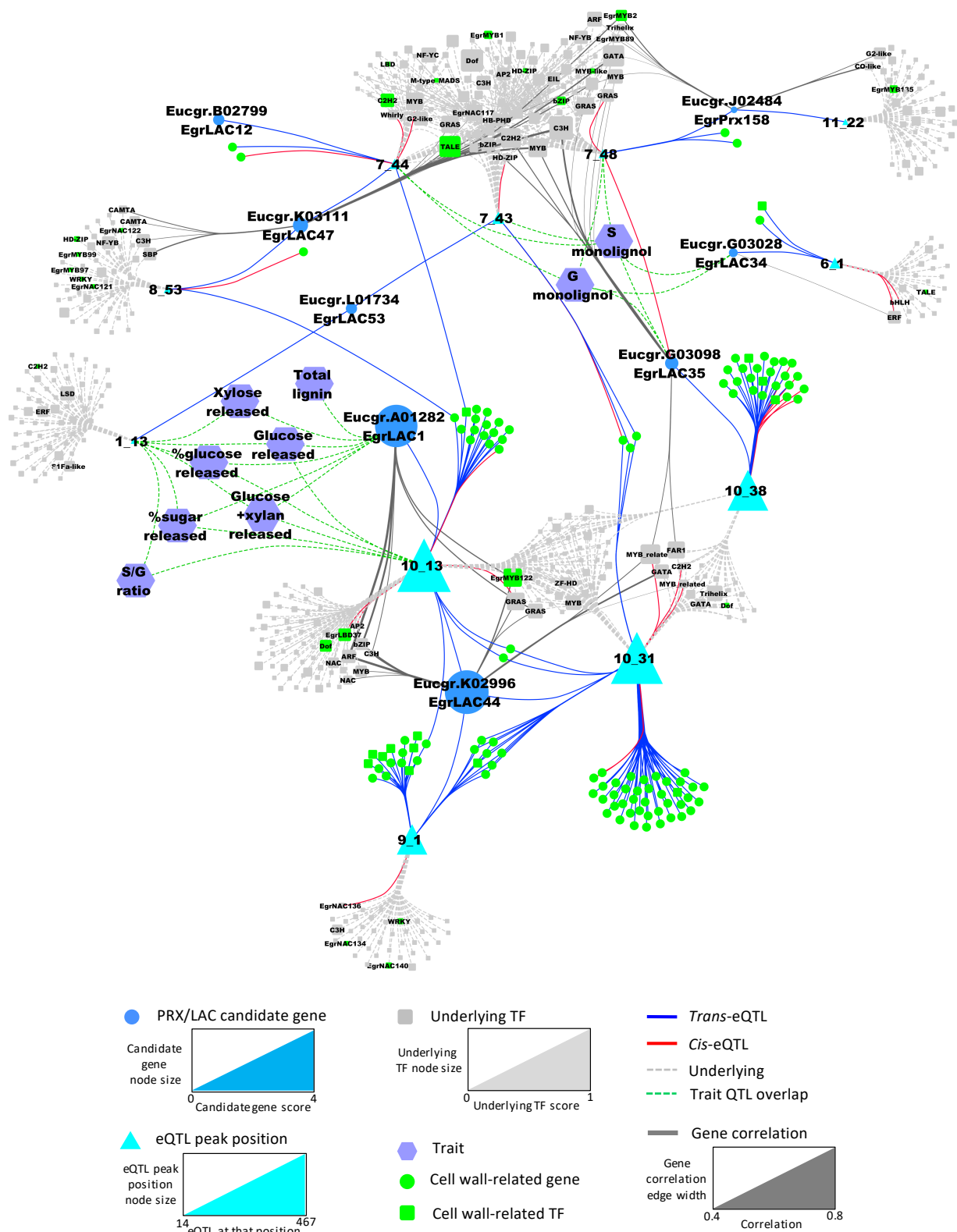

Supplement: Supplementary file 4 — Additional file 4: Figure S2. Systems genetics analysis of the eight LAC/PRX genes associated with secondary cell wall (SCW) biosynthesis in Eucalyptus xylem formation. The network was built from data exported from qtlXplorer and follows the same structure as presented in Fig. 6. Cis- and trans-eQTL associations connecting candidate genes (blue nodes) or cell wall-related genes (green nodes) to eQTL peak positions (turquoise triangles) are represented by red and blue edges, respectively. Transcription factors (TFs) underlying eQTL peak positions are represented by grey or green squares and connected to eQTL peak positions via grey dashed line edges. Gene expression profile correlations are represented by grey solid edges, with thickness proportional to the absolute value of the correlation. Physical overlap of trait QTLs (purple nodes) with candidate gene or eQTL peak positions are represented by green dashed lines. eQTL peak position node size is proportional to the number of genes having eQTLs (cis or trans) mapped at that genomic position. Underlying TF node size is proportional to the number and average value of the correlations of TFs with genes having cis- or trans-eQTLs mapped at that position (underlying TF score; Additional file 1: Table S8). Candidate gene node size is proportional to its score for prioritization (see Additional file 2: Methods S5; Additional file 1: Table S9), taking into account (i) their correlations (number and average value) with SCW genes across the population-wide transcriptomic data, (ii) the number of physical overlaps with candidate gene/SCW-related QTLs, (iii) the number of eQTLs mapped at SCW-enriched eQTL positions, (iv) the number of overlaps of their eQTL positions with SCW-related trait QTLs, and (v) the number of SCW-related TFs in the top 10 best candidate TFs underlying their eQTL positions. [file 12859_2021_4514_MOESM4_ESM.pdf]
